# What do we know about medical invalidation and related concepts? – A scoping review and thematic analysis about the definitions, measurements, causes, consequences and potential solutions for medical invalidation

**DOI:** 10.1186/s12913-026-14736-3

**Published:** 2026-09-08

**Authors:** Seraina Petra Lerch, Clara Stille

**Affiliations:** Department of Medical Psychology, University Medicine Greifswald, Walther-Rathenau-Str. 48, DE-17475 Greifswald, Germany

**Keywords:** Medical invalidation, Patient-provider-relationship, Medical gaslighting, Being taken seriously, Patient safety

## Abstract

**Background:**

Medical invalidation, medical gaslighting, and related constructs have gained visibility in public discourse but remain inconsistently defined in scientific literature. Despite growing research—often focused on specific diseases— to date, no single review has comprehensively synthesized their definitions, causes, consequences, or methods of measurement. This scoping review addresses this gap by examining medical invalidation and related constructs.

**Methods:**

Using a preregistered protocol, we systematically searched PubMed, CINAHL, Web of Science, Google Scholar, and ProQuest (dissertations) without year restrictions. Eligible sources included peer-reviewed empirical, theoretical, and conceptual work in English addressing invalidation, gaslighting, or closely related notions within healthcare. A total of 158 studies were identified through database searches and citation tracking. Data extraction followed a standardized schema, and findings were synthesized descriptively and through thematic analysis to clarify terminology, map determinants and outcomes, and identify existing measurement approaches.

**Results:**

The results showed substantial inconsistency in how “invalidation,” “not being taken seriously,” and “gaslighting” were defined. Medical invalidation emerged as a multifactorial phenomenon driven by diagnostic challenges, structural and societal factors, provider and patient characteristics, stigma, misattribution, interactional dynamics, academic knowledge gaps, and disease-related complexity. Invalidation was associated with wide-ranging behavioural, emotional, cognitive, physical, relational, and systemic harms, while validation had consistently beneficial effects. Proposed solutions in the summarized studies included communication improvements, clinician training, patient support, targeted research, and structural and systemic changes.

**Discussions:**

Medical invalidation represents a complex, systemic issue with significant implications for patient safety. The discussion highlights its multifactorial origins, its potential to cause both psychological and physical harm, and the need for clearer conceptualisation within the field. Advancing research requires validated instruments and longitudinal designs to examine underlying mechanisms and consequences. Addressing medical invalidation will demand multi-level interventions to improve communication, reduce structural barriers, and promote equitable, patient-centred care.

**OSF Preregistration:**

https://doi.org/10.17605/OSF.IO/MPE6U

**Supplementary Information:**

The online version contains supplementary material available at https://doi.org/10.1186/s12913-026-14736-3.

## Introduction

Research on medical gaslighting and medical invalidation has grown rapidly following increased coverage in mainstream media, reflected in a rising Google Trends interest since around 2018 and strong growth in 2020–2024 [1]. Evidence clearly indicates that a non-negligible proportion of patients with chronic conditions experience their symptoms being dismissed or not taken seriously, resulting in feelings of invalidation [2]. However, the terms medical gaslighting and medical invalidation remain underexplored within the realm of rigorous scientific inquiry. While initial peer-reviewed publications have begun to appear, the majority of studies focus on specific conditions, such as Long Covid [3], female-related health conditions [4], and Lyme disease [5].

In addition, numerous scientific commentaries [6, 7] and theoretical discussions [8, 9] have addressed the topic. The first systematic review examining the psychological impact of medical gaslighting on women has recently been published [10]. Furthermore, a recent systematic review summarized the consequences of invalidation, though it was restricted to 13 diseases [11]. A concept analysis of medical invalidation concludes, that the definitions and terms of medical invalidation, gaslighting and related concepts are still evolving and terminology is not clearly used [12]. Another review concludes that, although gaslighting and invalidation are conceptually distinct, the terms are often used interchangeably and inconsistently in the literature [13]. Consequently, there is a need for a comprehensive conceptualization and clear operational definitions of medical gaslighting and medical invalidation, as well as a systematic overview of their causes, consequences, potential interventions, and methods of measurement [13].

### Validation, invalidation, and gaslighting

If we want to understand and differentiate validation, invalidation, gaslighting, and related constructs within the medical context, it is necessary to first consider their general definitions, which are not specific to healthcare settings. As existing literature shows, Validation has been defined as “a way of communicating acceptance and understanding of another person’s emotions, thoughts, and behaviours” and as “a process in which a listener communicates that a person’s thoughts and feelings are understandable and legitimate” [14]. In contrast, invalidation has been conceptualized—primarily from a theoretical perspective—within ‘invalidating environments’ as described by Linehan, which (1) tell an individual that they are wrong in their description and analysis of their own lived experiences and (2) attribute an individual’s experiences to socially undesirable attributes [15].

Alongside the concepts of validation and invalidation, the phenomenon of medical gaslighting has also been described. Philosophical and theoretical accounts provide an important starting point for defining gaslighting. It has been characterized as a form of manipulation within an interpersonal relationship, in which a person or group sows seeds of doubt in an individual, leading them to question their own perceptions, judgment, feelings, or sanity [16]. In a similar vein, it has been described as manipulative behaviour that causes individuals to question their sanity or their perception of reality. These definitions suggest that invalidation and gaslighting share notable similarities, as in both cases an individual’s perceptions are questioned (see Table 1, first row). Gaslighting, however, extends beyond this by involving the active manipulation of a person’s reality [13, 17, 18]. Moreover, gaslighting tactics may include not only denial, dismissal, or minimization, but also lying, distortion, and blame shifting [13, 18]. Invalidation may serve as a precursor to or component of gaslighting [18]. Both phenomena may have serious consequences for affected individuals; however, their consequences remain unclear. To advance understanding of these phenomena, they can be examined through a socio-ecological perspective or by emphasizing the lack of patient-centred communication as a critical contributing factor.

### Invalidation through a socio-ecological lens

The patient–physician relationship is shaped not only by the individuals involved and their interpersonal dynamics, but also by broader contextual factors such as health care systems, policies, and continuity of care [19]. Thus, adopting a socio-ecological perspective highlights that invalidation is not solely the product of individual interactions but also embedded within larger structural conditions. This may help explain why invalidation occurs, as it is often unintentional and may arise from inadequate training or physician-related variables, while also being influenced by contextual conditions [11]. For instance, structural aspects of health care are known to foster implicit biases [20], which in turn can contribute to invalidation, particularly in time-limited or high-stress clinical environments [21]. Further research is needed to identify additional structural determinants of invalidation.

### Invalidation and patient-centered communication

While structural determinants play an important role, the quality of interaction and communication between patient and physician remains a critical contributing factor of invalidation. In recent decades, patient autonomy and patient-centered care have gained increasing prominence, with models of the patient–physician relationship shifting from a paternalistic approach to a patient-centered paradigm [22]. Patient-centered approaches encompass patient-centered communication (PCC) and a humanistic attitude. Invalidation directly contradicts the principles of PCC and a humanistic attitude [23]. According to Rogers, the physician’s attitude should embody acceptance, empathy, and congruence, expressed through open-ended questioning, verbalizing, paraphrasing, and active listening [24]. These reflective behaviours are frequently associated with validation [25]. Accordingly, integrating a humanistic attitude with patient-centred communication may potentially be essential for fostering a validating patient–physician relationship that may help prevent invalidation and gaslighting, although empirical evidence remains limited.

### Scoping review of different types of research and conceptual analysis

Even though there is a clear understanding of invalidation, validation and gaslighting outside of healthcare, those terms are inconsistent and diffuse used in the setting of health care [13, 25]. To accurately define and conceptualize medical invalidation and gaslighting, it is necessary to refine and differentiate both constructs and related constructs within the health care setting. The more widely researched term medical invalidation has been examined in a narrative review [25], which neither employed a systematic search nor incorporated related concepts. Although the term medical gaslighting is increasingly used in research, its application and definitions have been subject to criticism [13]; therefore, a clear conceptualization is required.

The present review addresses both concepts—medical invalidation, medical gaslighting—and thereby contributes to a more comprehensive understanding of patients’ experiences of not being taken seriously, a notion frequently used in the literature. Outside of healthcare, this experience is commonly understood as a form or consequence of invalidation; by examining it within the healthcare context, this review aims to clarify its conceptual boundaries and relationship to medical invalidation and gaslighting.

Despite emerging scholarship, there remains no systematic synthesis addressing the causes, consequences, and methodological approaches for studying medical invalidation. While a recent review systematically analysed the consequences of invalidation, it was restricted to specific diseases [11], and another review summarized behaviours and terminology associated with invalidation [25]. However, no comprehensive search has yet encompassed all terms related to invalidation, including gaslighting, across all diseases and contexts, nor has one synthesized evidence on causes, consequences, interventions, and measurement methods.

Given the breadth of the research question and the diversity of available evidence (qualitative, quantitative, commentary, and mixed-method studies), a scoping review is more appropriate than a systematic review [26], though a systematic search strategy can still be employed. Scoping reviews offer an overview and synthesis of a research field and, where appropriate, may incorporate conceptual analysis or thematic synthesis.

### Aim

Despite increasing attention to concepts such as medical invalidation and medical gaslighting, the field remains conceptually fragmented. Existing studies use inconsistent terminology, overlapping definitions, and diverse methodological approaches, making it difficult to compare findings or draw generalizable conclusions. Moreover, prior reviews have often focused on specific conditions or methodological approaches (e.g., qualitative studies), limiting the broader understanding of the phenomenon across healthcare contexts.

As a result, there is currently no comprehensive synthesis that (1) clarifies and distinguishes key concepts, (2) integrates both qualitative and quantitative evidence, (3) examines determinants and consequences across medical contexts, and (4) evaluates how these phenomena are measured. This lack of conceptual clarity and synthesis hampers the development of effective interventions and limits the ability to systematically study the phenomenon. To address these gaps, we conducted a pre-registered scoping review with the following aims.

The aim of this review is to synthesize the existing evidence on medical invalidation, medical gaslighting, and related concepts; to clarify and distinguish their definitions; and to evaluate the current state of research in order to identify knowledge gaps. For future research, consistent use of terminology is essential, making a conceptualization grounded in existing literature indispensable. Clear and consistent definitions are critical for identifying causes and consequences of medical gaslighting and invalidation, thereby informing the development of effective interventions. Furthermore, systematically compiling and evaluating current measurement methods is necessary to advance research in this field.

### Research questions

The primary research question is: What is medical invalidation and how can it be distinguished from related constructs such as medical gaslighting, and "not being taken seriously" in the medical encounter?

Further research questions are: 

What is the state of research of medical gaslighting, medical invalidation and related constructs?

What are the determinants for the occurrence of medical gaslighting/invalidation (e.g. bias, healthcare staff, healthcare system, diseases)?

What are the consequences of this phenomenon (e.g. psychological impact, somatic impact, costs, impact on healthcare)?

What are potential solutions to minimize the occurrence of these phenomena?

How is medical gaslighting/invalidation and related constructs measured in quantitative research and how is it coded in qualitative research?

## Methods

### Study design

A scoping review was performed based on the preregistration on the Open Science Framework under the registration number/name (quote). The scoping review checklist for preregistrations was applied [27]. The search was conducted on September 24th 2024.

### Inclusion criteria

Peer-reviewed empirical studies (qualitative, quantitative, or mixed-methods), peer-reviewed theoretical or conceptual articles, and relevant dissertations (ProQuest) were eligible if they examined medical gaslighting, medical invalidation, “(not) being taken seriously,” or close synonyms (e.g., dismissed, discounted, trivialized, discredited, disbelief of symptoms) within a health-care context. No publication year limits were applied to capture the conceptual development of these terms. All included literature had to be in English.

### Exclusion criteria

Papers not peer-reviewed or dissertations not officially accepted by a university were excluded. Further, abstracts were excluded and publications that had any other language than English.

### Information sources* and search strategy

The databases PubMed (via Ovid), CINAHL (via EBESCO), Web of Science and Google scholar were searched. For grey literature, dissertation and theses (via ProQuest) was searched to identify relevant dissertations. For each database, multiple search strategies were developed. The developed search concepts include medical gaslighting, medical invalidation, “(not) being taken seriously” in the medical setting and related concepts such as being discounted or dismissed. For Google Scholar, the first 50 results were screened, as reflected in the flow chart, which is a commonly applied approach in systematic reviews [28]. The detailed search string for all databases can be seen in the preregistration [29] or supplement 1.

After the first round of screening, for the preliminary relevant identified papers, were subject to both, forward and backwards citation searching. Contacting authors was not necessary.

### Selection of sources of evidence

In the first round, one reviewer screened all titles and abstracts. The first author independently verified a subset of these screening decisions as a second reviewer, with discrepancies discussed until at least 0.95 agreement was reached as measured by Cohen’s kappa coefficient, calculated using Covidence.

For the results of the forward- and backward citation searching, the same approach was applied. After that, a full text review was conducted for each text by two reviewers and discussed with the first author as third reviewer by disagreements.

Screener instructions included to identify if the article addresses medical gaslighting, invalidation or related constructs within the medical context. Further, it was clarified that only English articles will be included.

### Data charting process‡

To extract the relevant Data, Covidence was used. The data extraction form was developed by the first author. CS, CFAH, LRM, LB, and WR extracted data. All data extraction was verified by the first author SPL.

### Data items

Extracted data included, title, DOI, authors, country of research, type of article, research design, aim and if the papers main focus was medical Invalidation or related constructs or if it is just a by-product. Papers that had “not being taken seriously”, gaslighting or invalidation in the title, aim or research question were considered as a main focus, if only mentioned in the results but the research question had another focus, it was considered a by-product. Further we extracted population related variables, type of concept (e.g. medical gaslighting or invalidation etc.), definitions, conceptualizations or distinguishes, reasons for occurrence, consequences, potential solutions and measurement methods including surveys and quotes/themes for qualitative research.

### Critical appraisal of individual sources of evidence

Due to the heterogeneity of included evidence, no systematic appraisal was conducted. However, only peer-reviewed articles or dissertations that passed an evaluation process were included.

### Synthesis of results and conceptual analysis

To synthesize the results, both quantitative and qualitative approaches were considered. On one hand, descriptive analyses were conducted for year of study publication, country of study, type of publication, main focus of study, disease focus of the study, methodology and measurements and definitions. A thematic content analysis approach [30] were used for the reasons for occurrence, consequences and potential solutions. For this purpose, reported reasons for occurrence, consequences, and potential solutions were extracted from the studies and imported into MAXQDA. Themes were then inductively generated and refined based on the study data.

## Results

### Selection of sources of evidence

**Fig. 1:**
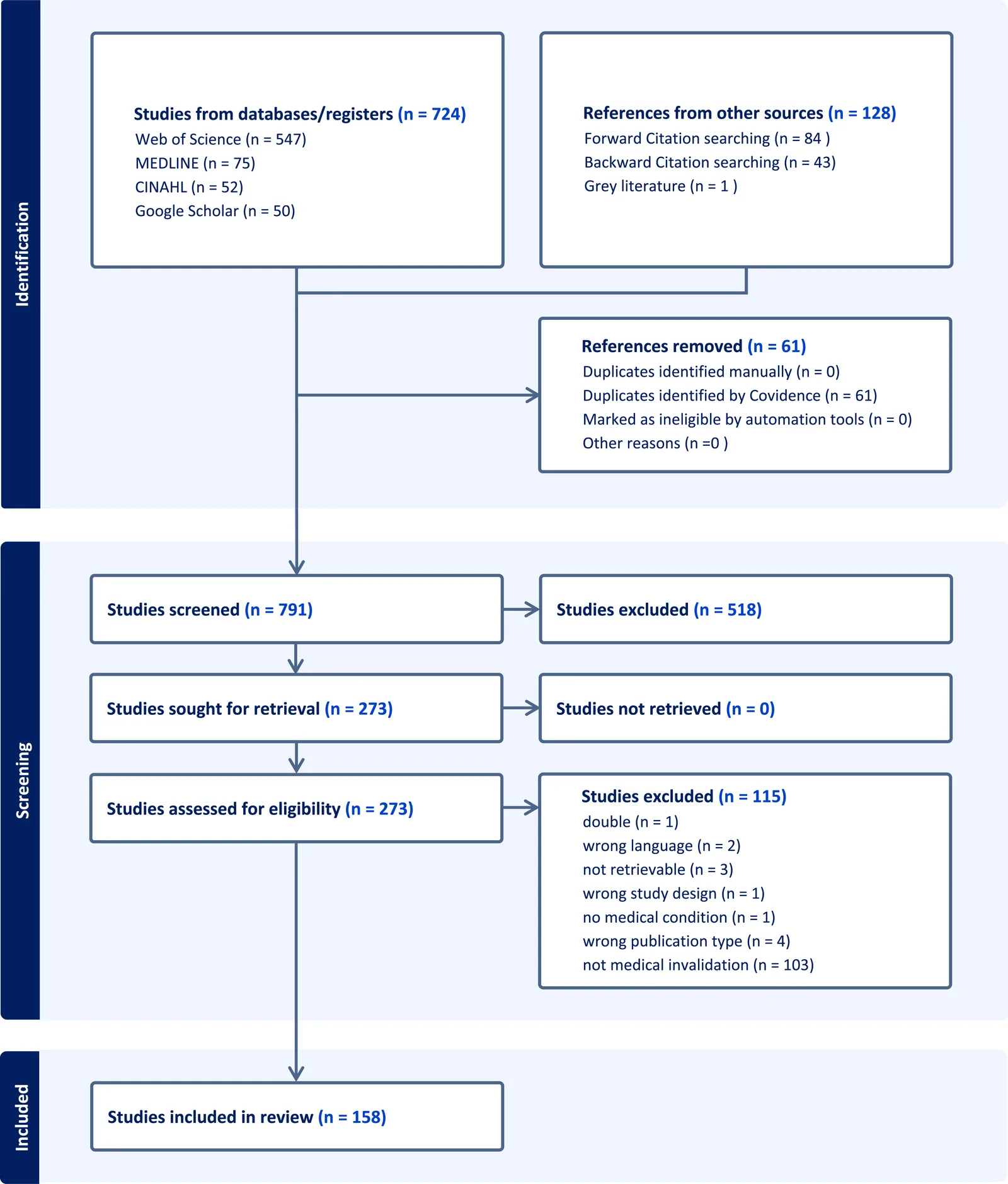
Flow chart of the review process

Within the first databases search, a total of 724 articles could be identified. Additional dissertation search as well as forward and backward citation searching of the 724 articles added another 128 articles. After duplicates removed (*n* = 61), a total of 791 studies were screened. 270 studies were assessed for eligibility with full text screening. In total, 158 studies were included. Detailed information can be seen on Fig. 1. An overview of all studies can be found in supplement 2.

### Characteristics of sources of evidence

Of the total 158 included studies, most of them were published the last 4 years (2021–2024), namely 93 (59%). The first identified study of the topic was from 1997. Most studies were either conducted in North America (*n* = 60%) or Europe (*n* = 58; 37%). Article types included original qualitative papers (*n* = 73; 46%), original quantitative papers (*n* = 35; 22%), reviews or meta-syntheses (*n* = 21; 13%), original mixed-method studies (*n* = 11; 7%) and opinion papers or commentaries (*n* = 18; 11%). In total, 81 studies (51%) had the main focus invalidation or related constructs and 77 (*n* = 48%) had another main focus. Within the main focus of invalidation or related topics, most studies actually did address invalidation (*n* = 38; 47%), followed by gaslighting (*n* = 21; 36%), not being taken seriously (*n* = 7; 9%) and both validation and invalidation together (*n* = 6; 7%). Within the studies in which invalidation was addressed but not the main focus, the primary topics were the general healthcare experiences (*n* = 30), diagnostic processes including delayed diagnosis (*n* = 12), the patient journey of a disease (*n* = 6), patient-physician relationship (*n* = 6), Stigma within healthcare (*n* = 5), patient safety (*n* = 3), implicit bias (*n* = 2), and epistemic justice (*n* = 2). Most studies addressed a specific disease or disease entity, including rheumatology (*n* = 12; 7%), psychiatric diseases (*n* = 9; 6%), gynaecological diseases (*n* = 16, 10%), cardiac diseases (*n* = 3; 2%), oncology (*n* = 3; 2%), neurological diseases (*n* = 7; 4%), internal medicine/infectiology (*n* = 4; 3%), disease entities such as general chronic illnesses (*n* = 3; 2%) and chronic pain (*n* = 28; 18%). Of all the included articles, only 40 (25%) provided a clear definition, 60 studies (*n* = 38%) an implicit definition and 58 studies (37%) provided no definition at all for invalidation or related constructs. A definition was classified as implicit when no explicit quotation, paraphrase, or referenced scientific source was provided, yet the meaning of the term was sufficiently clear from the context. Sources of invalidation were all sorts of healthcare professionals (HCPs), including all types of doctors, nurses, physiotherapists, psychologists, medical students, paramedics, and many others. Others sources, were family members, spouses, friends and general social network, social services, working colleagues and employers and one-self. An overview of these descriptive results can be found in Supplement 3.

**Fig. 2:**
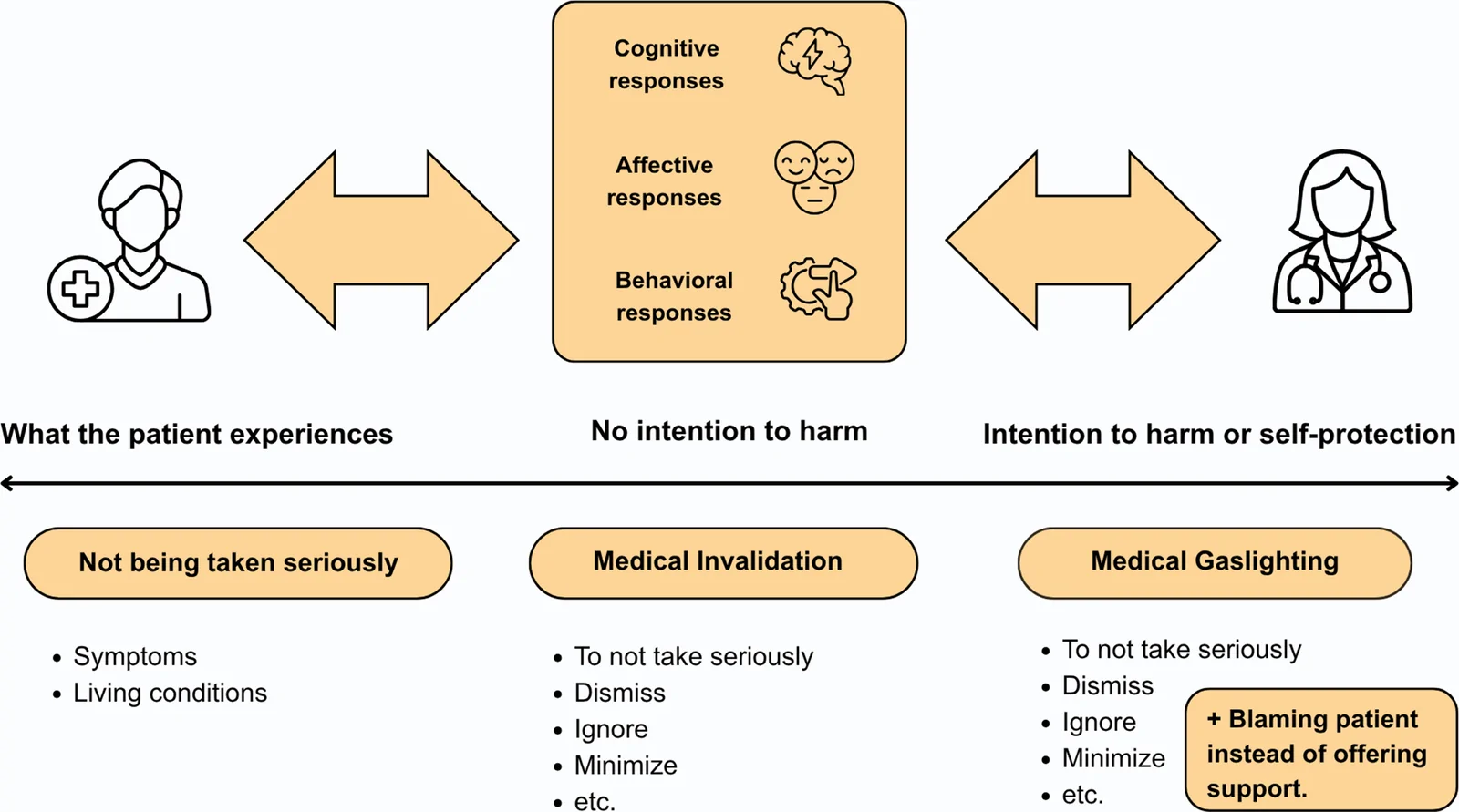
Synthesis of the concepts and distinctions of medical invalidation, medical gaslighting and “not being taken seriously” out of previous studies and theory

If definitions were provided, most studies (*n* = 17) referred either to Linehan’s definition of validation and invalidation [14] or within the medical context of Kool et al. (2010) [31]. The validation definition after Linehan includes “…a method, originally defined as a way of communicating acceptance and understanding of another person’s emotions, thoughts, and behaviours [14]. Further, validation was understood as “… a process in which a listener communicates that a person’s thoughts and feelings are understandable and legitimate.” For Linehan, “ invalidating environments (1) tell an individual that they are wrong in their description and analysis of their own lived experiences and (2) attribute an individual’s experiences to socially undesirable attributes” (see [15]). According to [31] invalidation to cognitive, affective, and behavioural responses of others that are perceived as denying, lecturing, overprotecting, not supporting, and not acknowledging the patient’s condition. In general, in the studies, invalidation referred to non-acceptance, misunderstanding, rejection, disbelief, discrediting, perceived misunderstanding or negative judgment by others, stigmatization, or suspicion that the problem is exaggerated or psychological. Not being taken seriously referred to both, if doctors did not adequately address medical symptoms, but also not the worries and concerns of those symptoms and the living worlds of patients [32], therefore referring to the patients experience rather than physician attributes. Gaslighting was understood “gaslighting as a form of manipulation within an interpersonal relationship in which a person or a group sows seeds of doubt in an individual, making them question their own perception, judgment, feelings and/or sanity [16]. While the behaviours and patient experiences associated with medical gaslighting and medical invalidation are often described using similar terms, a key distinction discussed in the literature relates to the underlying intent, with gaslighting implying a more deliberate or manipulative component. However, in the studies included in this review, intention was not assessed, suggesting that the reported phenomena may more accurately reflect medical invalidation rather than gaslighting. Figure 2 summarizes the concepts and distinctions and depicts the phenomenon across three dimensions arranged along a continuum with fluid transitions. Additionally, Table 1 (Row 2) summarizes how these concepts have been used in previous studies.

### Synthesis of results

#### Determinants of occurrence/causes

**Fig. 3:**
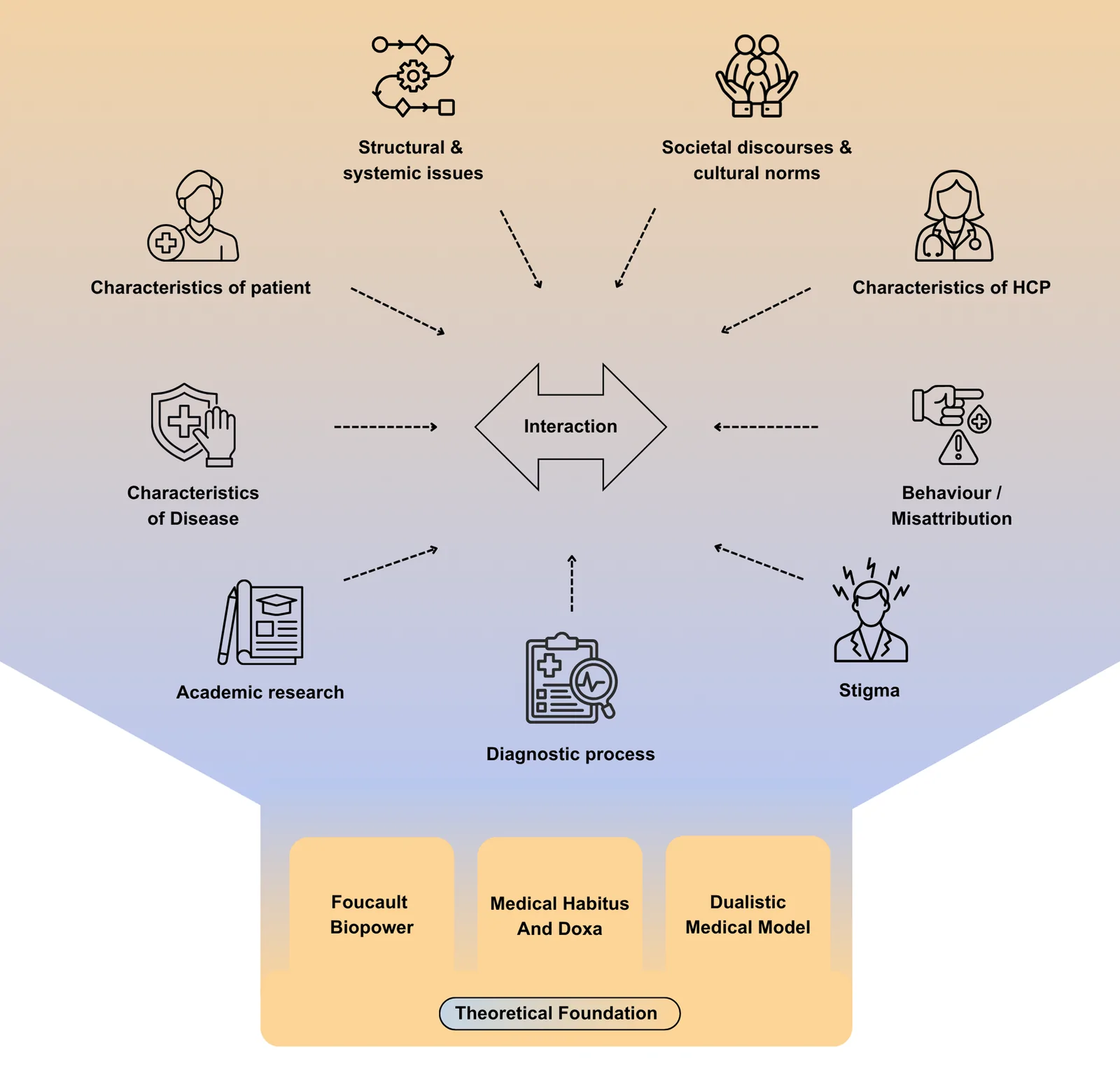
Synthesis of the determinants of medical invalidation

There are 10 main reasons that could be identified as determinants of medical invalidation. These reasons include the diagnostic process, societal discourses & cultural norms, structural/systemic issues, HCPs related variables, interaction of patient and HCPs, academic research, stigma, characteristics of the disease, characteristics of the patients, and characteristics of the HPC. One further category refers to theoretical explaining models of medical invalidation. These reasons are closely intertwined, and medical invalidation usually results from the combined influence of several overlapping factors rather than a single cause. Figure 3 illustrates the multi-level determinants of medical invalidation identified in this review. Supplement 4 summarizes the results as a table. There were 132 studies included for the thematic analysis of determinants of occurrence/causes [33–154].

#### Diagnostic process

Within the diagnostic process, there are several risk factors that might foster invalidation, for example general long diagnostic processes, missing of subjective and difficult to quantify symptoms within diagnostic criteria, lack of a formal diagnostic code, inconsistent diagnostic criteria, and lack of an objective diagnostic tests.

#### Societal discourses & cultural norms

Cultural expectations, for example that every illness should have a label and a treatment, and cultural injustice further contributes to medical invalidation. Societal pressures of idealism, meaning idealising doctors with “white coat authority” is a further contributor. The “opposite on the patient side, seeing people with intellectual disabilities as inferior, was a further theme. Further themes included paternalistic influences and policies, societal beliefs about severity thresholds (e.g. no suicide attempt means no access) and expectations of women.

#### Structural/systemic issues

Systemic contributors included economic burdens such as restraints by insurances or more administrative work leading to fewer time for patients. An increasingly unhealthy patient-population also contributes to increased need of resources and an economic burden. Further, as invalidation is mainly researched in the Western context, a western phenomenon was identified as a contributor: the systemic reductionism in western medicine. This refers to the privileging of objective, measurable data over subjective experience, often leading to the dismissal of patients’ self-reported symptoms. Further structural reasons include pressure on staff due to high workload and high demands of communication skills in certain settings. This is interrelated with a lack of resources within the system, lack of time, lack of training for both, competence and communication and lack of guidelines. Dependent on the healthcare system, there are health care access barriers, sometimes dependent on characteristics of the healthcare user, therefore interrelated with epistemic injustice.

#### Misattribution of symptoms

There is a risk of prematurely concluding that a mental illness is present when symptoms overlap, especially when personal characteristics are present or disease-specific characteristics are present.

#### Interaction of patients and HCPs

Interactional reasons for invalidation arise when communication and understanding break down between patient and provider. Limited dialogue, communication problems, and differing perspectives or health beliefs can prevent mutual understanding. Power imbalances and insufficient treatment offers further undermine patients’ sense of being heard. Frequent doctor visits or complex conditions may also lead clinicians to dismiss or minimize concerns. Together, these interactional dynamics create conditions in which patients feel misunderstood, disbelieved, or inadequately supported.

#### Academic research

In general, all under researched areas contribute to the problem of invalidation. For example, as there is little sex-specific research, there is also less knowledge about sex specific symptoms. This can contribute to the gender data gap and in the praxis, that symptoms are dismissed or similar due to a lack of knowledge. The same accounts for transgender health research or diseases that are not sufficiently researched.

#### Stigma

Different forms of stigma contribute to medical invalidation by shaping how patients’ accounts are perceived and valued. Implicit and unconscious biases can lead clinicians to discount symptoms that do not fit expected patterns, while social and interpersonal stigmas foster assumptions that certain groups exaggerate or misinterpret their experiences. Menstrual stigma, in particular, normalizes the minimization of pain and reinforces the belief that related symptoms are non-serious. Together, these stigmatizing processes reduce the credibility afforded to patients, increasing the likelihood that their concerns are dismissed or inadequately addressed.

#### Characteristics of the patients

Patient-related characteristics can also contribute to medical invalidation. Factors such as socioeconomic disadvantage, health-related vulnerabilities (e.g., substance use or weight-related challenges), and limited social support can reduce patients’ ability to navigate healthcare interactions and have their concerns taken seriously. Additionally, demographic characteristics and intersectional identities may shape how confidently patients present their symptoms or how they are perceived within clinical settings. Some of these factors overlap with processes of stigma, which can further influence clinical perceptions and interactions. Collectively, these elements affect whether patients’ reports are acknowledged, understood, or unintentionally minimized.

#### Characteristics of the disease

Disease-related characteristics play a central role in shaping the likelihood of medical invalidation. Conditions presenting with complex, atypical, ambiguous, or medically unexplained symptoms often challenge diagnostic processes, increasing the risk that clinicians minimize or dismiss patient reports. Similarly, diseases that manifest with psychological comorbidities or psychological features are frequently misattributed to mental causes, leading to premature closure and insufficient biomedical investigation. Patients with chronic, multifaceted, or poorly understood conditions—including autoimmune, pain-related, gynaecological, and functional disorders—face heightened invalidation because limited clinical knowledge undermines providers’ confidence in interpreting symptoms. Moreover, when symptoms are not approached holistically, fragmented care can obscure underlying patterns and further reinforce doubt. Overall, insufficient understanding, diagnostic complexity, and symptom ambiguity constitute key disease-related drivers of medical invalidation.

#### Characteristics of the people not taking seriously

#### Characteristics of HCP

Healthcare professional–related characteristics also shape the occurrence of medical invalidation. Limited time, insufficient preparedness, or discomfort during clinical conversations can lead to incomplete assessments and premature dismissal of patient concerns. Additionally, the HCP’s general attitude and the influence of personal moral beliefs may affect how seriously patients’ symptoms are taken. Together, these factors can hinder open communication and reduce the likelihood that patients’ experiences are fully heard and validated.

In the identified studies, the identified HCP that conducted invalidation were primary care physicians, all types of specialist physicians, nurses, psychotherapists, paramedics, midwives and physiotherapists.

#### Theoretical explanation models

The studies included in the review identified three theoretical models that help explain why medical invalidation occurs. Foucauldian biopower highlights how medical authority enforces normative expectations, making atypical experiences more likely to be dismissed. Medical habitus and doxa illustrate how clinicians’ internalized assumptions shape perceptions of credible symptoms and patients. The dualistic medical model further contributes by prioritizing physical evidence and discounting symptoms without clear biological markers. Together, these frameworks illuminate structural and epistemic mechanisms underlying invalidation.

### Consequences of invalidation

Across the included studies, nine overarching themes of consequences were identified. Medical invalidation was associated with behavioral, affective/emotional, and cognitive consequences, and further impacted patient satisfaction, mental and physical health, social relationships, and the patient–provider relationship. In addition, broader global consequences were reported. There were 144 studies included for the thematic analysis of the consequences of invalidation [33–57, 59–66], 68– [4, 103–106, 108, 110–123, 125–145, 147–172].

**Fig. 4:**
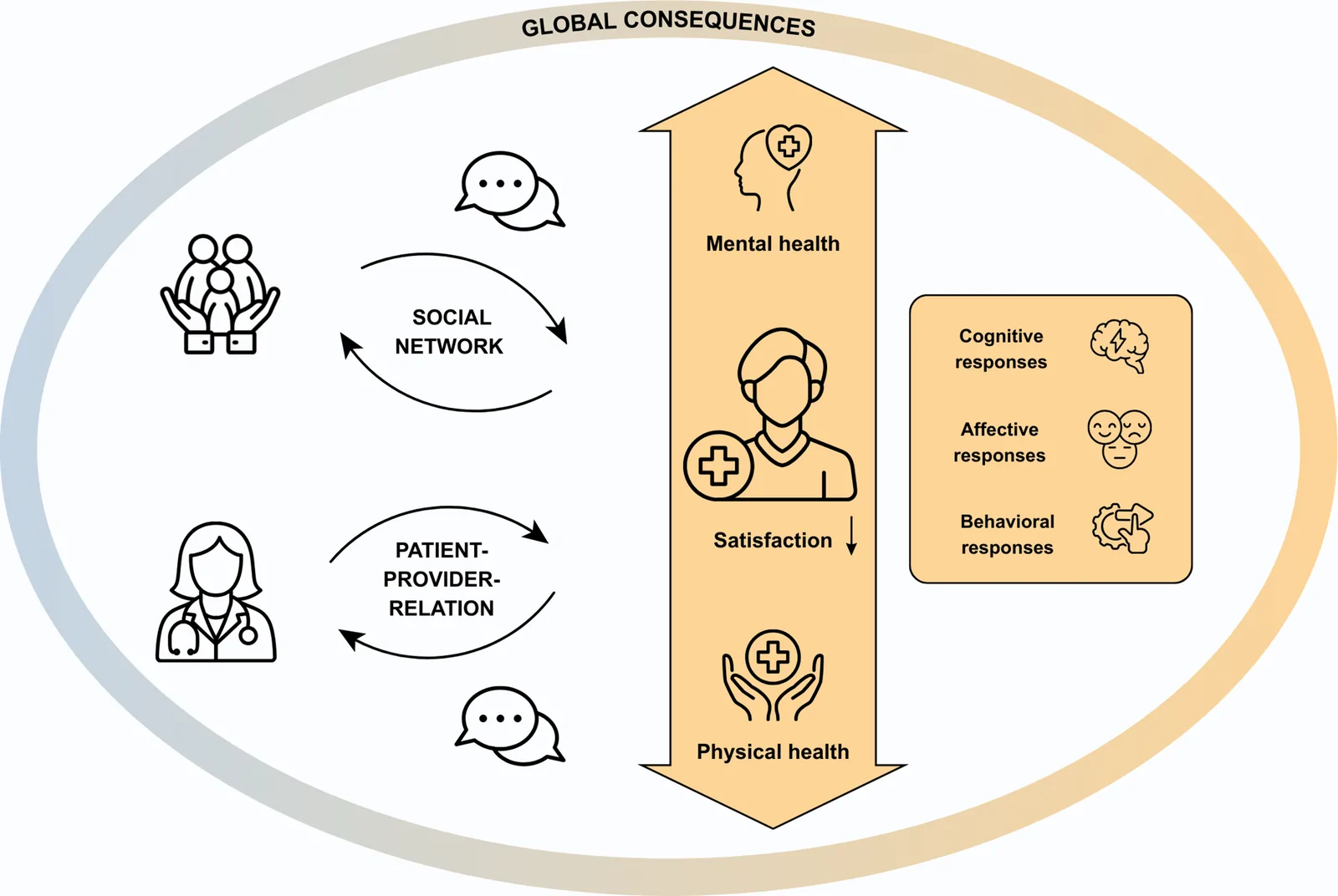
Synthesis of the consequences of medical invalidation

As illustrated in Fig. 4, these consequences are not presented as a linear or causal model but as a conceptual mapping that synthesizes the breadth and systemic nature of reported outcomes. The figure highlights how consequences may co-occur and interact across multiple domains, rather than unfolding in a unidirectional sequence. Central to this mapping is the patient–provider interaction, which appears to play a key mediating role by shaping how invalidation is experienced, interpreted, and translated into downstream emotional, behavioral, and health-related outcomes. A tabular overview is provided in Supplement 5.

#### Behavioural consequences of invalidation

Behavioural consequences included decreased help seeking, non-adherence to treatment, exaggerating symptoms and self-educative behaviour.

#### Affective/emotional consequences of invalidationAffective/emotional consequences of invalidation

Affective and emotional consequences were reported as short-term consequences to invalidation. Therefore, it is distinct from “mental health consequences” that describes more long-term consequences to invalidation.

These consequences included feelings including but not exhaustively anger, frustration, sadness, feelings of inadequacy, shame, grief and embitterment. Other negative emotional consequences included emotional dysregulation, learned helplessness, and emotional distress in general.

#### Cognitive consequences of invalidation

Cognitive consequences included increased rumination and worry, a negative body-image and in general an erosion in self-concept, a loss of self-confidence, self-doubt and decreased health-literacy. Further, invalidation fostered a deeper belief in non-scientific explanations of disease and treatment.

#### Consequences on satisfaction

When experiencing invalidation, patients reported a decreased satisfaction with healthcare.

#### Physical health consequences

Experiencing invalidation was associated with various physical health consequences including a general physical distress, increased symptoms often due to a lack of treatment, decreased life expectancy, especially when treatment then followed to late or a misdiagnosis, relapses, medical complications up to close to death experience when a severe condition was identified to late and a general worsened health.

#### Mental health consequences

In long-term mental health consequences, a trauma experience was prevalent. This included both, general traumatic distress without a post-traumatic stress disorder as well as post-traumatic stress disorder. Other long-term mental health consequences included persistent feelings and experiencing of depression, anxiety, and stress. In general, long term mental health did suffer with a worsened psychological sate after experiencing invalidation. Some patients who have experienced invalidation have developed suicidal thoughts.

#### Consequences for social relationships

Invalidation led to loneliness, social exclusion and isolation. Further, patients received decreased social support for coping with their illness after being invalidated by the HPC. This was suspected to be as the social environment took up the invalidation of the HPC and the patient was seen as non-credible. The same accounts for romantic couple relationships, where patients experienced more challenges, for example reduced empathy.

#### Consequences for health care and relationship with HCPs

There were general consequences for both, general for health services and the relationship with the HCP. For example, patients started to strive for proof within the relationship with the HCP. Further, they lost trust in HCP and the health care system. Generally, the relationship with the HCP was impaired, for example through negative interaction cycles.

Consequences for health services included an increased dependence on medical care as well as either delayed or increased support seeking but also seeking support outside the health care system. Invalidation was further associated with unnecessary medical interventions, meaning a qualitative worsened health care. Further it was associated with prolonged diagnostic processes, delayed treatment, and undertreatment and misdiagnosis.

#### Global consequences

Global consequences included loss of trust in government or welfware denial. Further, it had financial consequences on several levels, as patients had delayed treatment, wrong treatment or worsened condition. Financial consequences could also come from employer consequences when patients were more absent or reduced work hours due to a worsened condition or more doctor visits.

### Consequences of validation

Validation had positive consequences for the patient’s behaviour, emotions, and cognitions. Further it positively impacted patients’ satisfaction, physical health, the relationship with HCP and health services. The consequences of validation are summarized in supplement 5.

#### Behavioural consequences of validation

Behavioural consequences of validation are that the patient is empowered and acts on behalf of himself. Further, it fosters adherence to treatment.

#### Affective/emotional consequences of validation

Validation had positive emotional and affective consequences for patient, including feeling safe, understood, humanized and respected. It also decreased unpleasant feelings such as anger and frustration. When patient had a negative affect before the health care visit, it decreased the negative effect. If they already had a positive affect, this positive affect was maintained.

#### Cognitive consequences of validation

Patients experiencing validation had an improved symptom comprehension, which then was also associated with the behavioural consequences of adherence. They further reported enhanced self-worth and decreased worry.

#### Consequences on satisfaction

Patients experiencing validation report both, a greater satisfaction with treatment and greater health-related quality of life satisfaction.

#### Physical health consequences

Known physical health consequences of validation are overall better treatment outcomes and decreased pain.

#### Consequences for health care and relationship with HCPs

Patients experiencing validation reported both, increased trust in their HCP and the healthcare system. Patients further were less entitled to health care support and were more likely to disclose symptoms or relevant information towards their HCP.

### Potential solutions

Potential solutions can be found on different levels: Some solutions address the HCP, others the patient-provider interaction, some the patient. There are global proposed solutions on the level of the healthcare system, general structural solutions, and solutions on the research level. Potential solutions are summarized in supplement 6. There were 140 studies included for the thematic analysis of the potential solutions [33–55, 57–76, 78–86, 88–94, 95], [4, 103–120, 122], [32, 161, 163, 165, 167, 168, 170, 171, 173–176].

#### Solutions on the level of the HCP

Identified solutions on the level of the HCP included to foster openness to emotional ambivalence and uncertainty as well as professional conducts and ethics. HCP should be willing and taught to admit knowledge gaps and refer patients appropriately. Further to educate HCP on respective diseases is essential. To tackle down the phenomena of invalidation, fostering interpersonal and communication skills in HCP is necessary. Communication skills include empathetic and open communication as well as non-verbal communication.

#### Solutions on the level of patient-provider interactionSolutions on the level of patient-provider interaction

With the patient-provider interaction, it is essential that a HCP provides clear guidance on the next steps. Further, these next steps need to be developed and decided on in a participatory way, making shared-decision making and participatory communication essential.

#### Solutions on the level of the patient

We identified solutions on the patient level. Some of these solutions sometimes only apply if patients have already experienced invalidation to prevent them from further invalidation or to help patients cope with the experience. Helping patients cope with the experience can further prevent invalidation, as one of the consequences of invalidation are negative interaction cycles.

One potential solution on the patient level is to provide space to patients where they can discuss their experiences. This can happen but not exclusively through online support group and communities, that are helpful, too. Patients can further be encouraged that they take social support with them to the appointment. Another strategy of patients that can be fostered is to let them track their symptoms and document their interactions and steps with each HCP visit. Fostering the skill of “standing up for oneself” can also be supportive in patients. Patients should further be encouraged to search for a doctor that takes them seriously if possible (this is sometimes restricted through structural aspects) or seek a second opinion. Patients can also be instructed to anticipate dismissal and to disclose symptoms strategically.

#### Solutions for the health care system

Some potential solutions address the health care system and include individualized health care. Health care should be individualized and tailored for individual patients. Within the system, there should be multi-disciplinary treatment options, including complementary and alternative medicine. This is especially important in ambiguous, uncertain, and complex situations. The health care system should be patient-centered and apply a holistic and bio-psycho-social approach. Further it should be standard to raise awareness towards implicit bias and patients should be protected against discrimination. Health care access threshold should be low, ensuring all patients have access to care. Continuity of care should be ensured, which includes transitional health care from youth into adult care.

#### Structural solutions

Structural solutions include the diversification of HCP, fostering narrative medicine, apply approaches that lead to destigmatization, combat health inequalities, and to standardize the regular review and implementation of treatment and diagnostic guidelines. Further, the health care system should be adequately founded to increase specialist capacity, ensure needed diagnostic tests are provided by insurance and that the HCP has sufficient time.

#### Solutions on the level of research

Research should include sex and gender data to ensure there is sufficient knowledge on sex-and gender specific symptoms. Further, unexplained medical phenomena should be research, to have better understanding. Better understanding and knowledge can help prevent invalidation.

### Measurement

Original papers included qualitative approaches (*n* = 73; 46%), original quantitative papers (n = and original mixed-method studies (*n* = 11; 7%). Within the 35 quantitative studies, 12 (34.3%) used the Illness Invalidation Inventory (3*I) [31] in full, and 5 (14.3%) used one of the five subscales, and a further study (2,9%) modified the 3*I. Three studies used the validating and invalidating response scale (VIRS) [177] (8,6%). Two studies (5,7%) designed their used questions self (e.g. if they have ever felt a HCP has dismissed or ignored their symptoms). One study (2,9%) used the subscale Feelings of Medical Profession (FoMP) of the Endometriosis Health Profile Modular Questionnaire (EHP-30) [178] and another (2,9%) the Barrett-Lennard Relationship Inventory (BLRI) [179]. Unfortunately, 10 studies (28,6%) did not specify their used questionnaire.

## Discussion

### Summary of evidence

Of the total 158 included studies, most of them were published the last 4 years (2021–2024), namely 93 (59%). This highlights a recent surge of interest in the topic. The observed increase in research is likely multifactorial and may be associated with the rise of long COVID [3] and similar conditions following the pandemic, as well as the growing influence of social media, which has amplified patients’ ability to have their voices heard [166]. However, there is currently no clear evidence explaining why research on this topic has grown so rapidly.

A considerable variation could be identified in the use of terms for “invalidation”, “not being taken seriously”, and “gaslighting”, with many studies offering only implicit or no definitions. Our review highlights that medical invalidation arises from overlapping factors including diagnostic challenges, structural pressures, societal norms, provider characteristics, patient characteristics, stigma, misattribution of symptoms, patient–provider interaction, academic research gaps, and disease-related factors. Invalidation was linked to extensive behavioural, emotional, cognitive, physical, relational, and systemic harms, whereas validation produced consistently positive outcomes. Proposed solutions span multiple levels, including improved clinical communication, better training, enhanced patient support, structural reforms, and targeted research to address knowledge gaps and reduce the risk of invalidation across healthcare settings.

Our analysis revealed that medical gaslighting and medical invalidation, “not being taken seriously” and related constructs were often insufficiently defined in studies. These studies that defined the constructs for invalidation often included experiences such as not being taken seriously, dismissal, and minimization in their analyses, indicating that concepts such as not being taken seriously, dismissing, minimizing, disbelieving, and ignoring can be understood as different forms of medical invalidation, in line previous research [180]. At the same time, the literature reflects two complementary but distinct ways of organizing these concepts. On the one hand, the conceptualization described above frames “not being taken seriously” as a subdimension of invalidation. On the other hand, an experiential or perspective-based logic distinguishes between patient-reported experiences (e.g., not being taken seriously) and invalidation as a construct rooted in HCPs cognitions, behaviors, and attitudes. In this sense, while the aforementioned concepts primarily capture what patients experience, invalidation more often refers to the cognitive, behavioral, and attitudinal processes of HCPs. Additionally, the literature distinguishes between illness invalidation and medical invalidation. While illness invalidation refers primarily to the invalidation of patients’ symptoms, medical invalidation encompasses a broader range of experiences, including aspects of patients’ lived worlds beyond symptom presentation. Accordingly, medical invalidation represents the more encompassing concept. Medical gaslighting often used definitions that were in line with the definitions of medical invalidation, without distincting it. Further, those definitions often did not include, that the gaslighting person did the gaslighting on purpose, which would be a prerequisite of the original gaslighting definition [16]. Therefore, medical invalidation seems to be the more appropriate (umbrella) term for most clinical and research contexts in which intentional manipulation cannot be clearly established. Medical gaslighting should be reserved for cases in which deliberate manipulation is evident. This is line with recent reviews and conceptualization approaches on medical invalidation [12, 13]. Generally, not being taken seriously referred to the patient experiences, whereas invalidation and gaslighting referred to the other party´s behaviour, affections or cognitions. For invalidation, these behaviours included for example but not exhaustively dismissing symptoms, minimizing them and ignore them, in line with a recent conceptualization on medical invalidation [12]. These could also refer to gaslighting if the behaviour is intentionally conducted to harm somebody or to protect oneself, for example when patients are blamed for their symptoms to cover up that there is no support provided.

This still insufficient conceptualization may contribute to the predominance of qualitative research in the field. Numerous commentaries exist on the topic, yet quantitative evidence remains limited. Consequently, many hypotheses and unexamined assumptions persist without empirical support. The research area would benefit from the development of validated instruments to assess different dimensions of (in)validation at both the patient and HCPs levels. Once such measures are established, longitudinal studies could begin to examine the causal consequences of invalidation, which to date have been explored primarily through qualitative or cross-sectional designs. Irrespective of study design, future research should explicitly consider the position along the proposed continuum that is being addressed. Given the fluid boundaries and conceptual overlap between related constructs, it is essential that studies clearly define the terminology used and specify which aspects or dimensions of (in)validation are being examined. Such conceptual transparency is crucial to improve comparability across studies and to advance cumulative knowledge in the field.

Moreover, invalidation has largely been studied within the context of specific diseases. Investigating the phenomenon across conditions could help identify shared mechanisms underlying its occurrence and its consequences. In addition, the perspectives of HCPs have so far been insufficiently considered; incorporating their viewpoints could offer valuable insights and should not be overlooked. Finally, representative studies are lacking that could substantiate gender-based and minority-related hypotheses regarding the occurrence of invalidation.

The occurrence of medical invalidation can be attributed to a complex interplay of factors, including challenges in the diagnostic process, societal discourses and cultural norms, structural and systemic healthcare issues, interactional dynamics between patients and HCPs, underrepresentation in academic research, stigma and implicit biases, disease- and patient-related characteristics, as well as healthcare professional-related variables and overarching theoretical frameworks.

These determinants are deeply interrelated and often mutually reinforcing, meaning that medical invalidation rarely stems from a single cause. Rather, it typically results from the convergence of multiple contributing factors. This multifactorial nature can be aptly illustrated by the “Swiss cheese model” used in public health, where various systemic vulnerabilities or individual-level barriers—each represented as a slice with holes—must align for invalidation to occur, highlighting the cumulative and systemic nature of the phenomenon [181, 182].

The Swiss cheese model further illustrates that severe consequences of medical invalidation—such as long-term negative mental health outcomes or significant physical harm—typically arise only when multiple contributing factors align, rather than from invalidation alone [16]. When additional vulnerabilities or systemic gaps coincide, the cumulative impact can lead to substantial psychological distress. The most frequently reported mental health consequences were trauma-related symptoms, particularly when invalidation resulted in physical harm or when its negative effects persisted over time. This pattern aligns with existing research on medical trauma, which suggests that medical invalidation may be a precursor to, or a contributor to, such experiences [183, 184]. Consequently, medical invalidation could be considered a factor that increases the likelihood of experiencing medical trauma. However, not everyone experiences such severe consequences. These consequences can be short-term, affecting emotions, behaviour, and cognition or the relationship with the provider. For some, the effects are more short-term, influencing emotions, behaviour, cognition, or the patient–provider relationship. Even in less severe cases, medical invalidation consistently produces psychological harm. Within patient safety frameworks, these forms of psychological harm are recognised as legitimate safety concerns [185], and several documented sources and contributors to patient harm overlap conceptually with drivers of medical invalidation [186]. Accordingly, medical invalidation can be understood not only as a form of psychological harm but also as a potential source of physical harm.

### Limitations

Because most studies were qualitative and no longitudinal studies were identified, the reasons, consequences, and potential solutions discussed cannot be interpreted causally. Nevertheless, this review provides a comprehensive overview of the current understanding of medical invalidation, which can inform and guide future empirical investigations.

The included studies were highly heterogeneous, rendering a formal risk-of-bias assessment unfeasible. However, this heterogeneity aligns with the aim of the review, which was to map and synthesise the existing literature on the topic in its current breadth.

## Conclusions

This scoping review demonstrates that medical invalidation is a multifaceted phenomenon arising from intersecting individual, interpersonal, structural, and epistemic factors. Its consequences range from short-term emotional and cognitive distress to long-term psychological and physical harm, underscoring its relevance as a patient safety issue. Clear conceptual definitions, validated measurement tools, and longitudinal research are critically needed to advance the field. Addressing medical invalidation requires interventions at multiple levels—clinical communication, training, structural reform, and research—to ensure that patients’ experiences are recognised, taken seriously, and integrated into equitable, patient-centred care.

**Table 1:** Synthesis of the concepts and distinctions of medical invalidation, medical gaslighting and “not being taken seriously” in theory, previous studies and recommendations

	Not being taken seriously	Related constructs	Illness Invalidation	Medical Invalidation	Medical Gaslighting
Operationalized Definition	This term describes the patient’s experience of not being taken seriously. However, it may also reflect healthcare professional (HCP) behaviour.	This category summarizes terms describing concrete HCP behaviours that invalidate patients, including (but not limited to) ignoring, dismissing, not believing, discounting, or trivializing. However, it may also reflect the patients experiences.	HCP behaviours, attitudes, or cognitions toward patients regarding their symptoms, which communicate to the patient that their descriptions are incorrect or attribute these experiences to undesirable personal characteristics.	HCP behaviours, attitudes, or cognitions toward patients regarding their symptoms, worries, concerns, or lived world, which communicate to the patient that their descriptions are incorrect or attribute these experiences to undesirable personal characteristics.	Manipulative and intentional behaviours and communication by an HCP toward a patient regarding their symptoms, worries, concerns, or lived world, which communicate to the patient that their descriptions are incorrect or attribute these experiences to undesirable personal characteristics.
Previous use in studies	This term has been used to describe a patient experience.	These terms are usually used exclusively to describe patient experiences rather than HCP behaviours or attitudes.	In the presented studies, patients’ perspectives were included, focusing on the behaviours, cognitions, and affects they observed in HCPs, rather than on concrete HCP behaviours.	In the presented studies, patients’ perspectives were included, focusing on the behaviours, cognitions, and affects they observed in HCPs to describe medical invalidation, rather than concrete HCP behaviours.	Medical gaslighting has been used to describe the patient experience of not being taken seriously. No studies have measured this construct at the HCP level.
Conclusion/Recommendation	This refers to a patient experience in which symptoms, concerns, or the patient’s lived experience are invalidated within a medical context. It may also describe healthcare professional (HCP) behaviour that invalidates these experiences.	These constructs describe both patient experiences and HCP behaviours that invalidate patients’ symptoms, concerns, or lived experiences.	Illness invalidation is an umbrella term encompassing multiple HCP behaviours, attitudes, and cognitions (including those listed under “related constructs”) toward patients’ illness-related symptoms. At the same time, it also serves as an umbrella term summarizing patients’ experiences.	Medical invalidation is an umbrella term encompassing multiple HCP behaviours, attitudes, and cognitions (including those listed under “related constructs”) toward patients’ symptoms, concerns, and lived world. At the same time, it also serves as an umbrella term summarizing patients’ experiences.	Although, by definition, gaslighting requires intentionality on the part of the HCP, this has not been operationalized in studies. In practice, when studies refer to medical gaslighting, they often describe medical invalidation.

## Electronic Supplementary Material

Below is the link to the electronic supplementary material.

Supplementary Material 1

Supplementary Material 2

Supplementary Material 3

Supplementary Material 4

Supplementary Material 5

Supplementary Material 6

## Data Availability

Extracted data of our review is available as supplementary material.
