# Supplementary material for: What do we know about medical invalidation and related concepts? – A scoping review and thematic analysis about the definitions, measurements, causes, consequences and potential solutions for medical invalidation
Source: BMC Health Serv Res. 2026 Sep 8;26:1252. doi: 10.1186/s12913-026-14736-3 (PMC13560384; doi:10.1186/s12913-026-14736-3)
Supplement: Supplementary file 1 [file 12913_2026_14736_MOESM1_ESM.docx]

**Supplement 1 – Search Strings**

MEDLINE (Pubmed via Ovid) for medical gaslighting:

Medical gaslighting.mp

(note that the keywords search gaslighting( and professional-patient Relations/ combined gave 0 searches while medical gaslighting.mp gave 14 hits on june 7th)

MEDLINE (Pubmed via Ovid) for medical invalidation:

Medical invalidation.mp

(note that there is no indexed word for invalidation or medical invalidation)

MEDLINE (Pubmed via Ovid) for (not) being taken seriously:

3	Professional-Patient Relations/	28741

6	being taken seriously.mp. 112

7	3 and 6	6

MEDLINE (Pubmed via Ovid) for related constructs:

3	Professional-Patient Relations/	28741

11	((dismiss* or devalu* or trivial* or reject* or normal* or ignor* or disqual* or discred* or disbelief* or deligitim*) and symptoms).ab,kf,kw,ti. 107544

12	3 and 11	47

CINAHL on medical gaslighting

S1	medical gaslighting	Expanders - Apply equivalent subjects

Search modes - Proximity	Interface - EBSCOhost Research Databases

Search Screen - Advanced Search

Database - CINAHL Complete	9

CINAHL on medical invalidation

invalidation AND patient physician relationship	Expanders - Apply equivalent subjects

Search modes - Proximity	Interface - EBSCOhost Research Databases

Search Screen - Advanced Search

Database - CINAHL Complete	2

CINAHL on “not being taken seriously”

taken seriously AND patient-physician	Expanders - Apply equivalent subjects

Search modes - Proximity	Interface - EBSCOhost Research Databases

Search Screen - Advanced Search

Database - CINAHL Complete	18

CINAHL for related constructs

S7	dismiss OR devalue OR trivialization OR reject OR normal OR ignore OR disqualification OR discredited OR disbelief OR delegitimization	Expanders - Apply equivalent subjects

Search modes - Proximity	Interface - EBSCOhost Research Databases

Search Screen - Advanced Search

Database - CINAHL Complete	228,201

S12	symptoms	Expanders - Apply equivalent subjects

Search modes - Proximity	Interface - EBSCOhost Research Databases

Search Screen - Advanced Search

Database - CINAHL Complete	432,722

S14	(patient-physician) AND (S7 AND S12 )	Expanders - Apply equivalent subjects

Search modes - Proximity	Interface - EBSCOhost Research Databases

Search Screen - Advanced Search

Database - CINAHL Complete	22

Web of Science on medical gaslighting

ALL=(medical gaslighting) (36)

Web of Science on medical invalidation

ALL=(medical invalidation) (441)

Web of Science on “not being taken seriously”

(ALL=(being taken serious)) AND ALL=(patient physician relationship) (10)

Web of Science for related constructs

((((((((((AB=(dismiss*)) OR AB=(devalu*)) OR AB=(reject*)) OR AB=(normal*)) OR AB=(ignor*)) OR AB=(disqual*)) OR AB=(discred*)) OR AB=(disbelie*))) AND AB =(medical symptoms)) AND AB =(patient physician relationship) (42)
