# Supplementary material for: What do we know about medical invalidation and related concepts? – A scoping review and thematic analysis about the definitions, measurements, causes, consequences and potential solutions for medical invalidation
Source: BMC Health Serv Res. 2026 Sep 8;26:1252. doi: 10.1186/s12913-026-14736-3 (PMC13560384; doi:10.1186/s12913-026-14736-3)
Supplement: Supplementary file 2 [file 12913_2026_14736_MOESM2_ESM.docx]

| **Mainthemes** | **Subthemes** | **Subsubthemes** | **Description** |
| --- | --- | --- | --- |
| **Solutions on the level of the HCP** | Foster HPCs openness to emotional ambivalence and uncertainty |  | As invalidation occurs in the context of medical uncertainty and ambuigance, fostering openness to uncertainty and ambivalence in HCP is necessary. |
|  | Admitting knowledge gaps and refer appropriately |  | One concrete strategy to deal with uncertainty or not knowing that helps preventing invalidation is the HCP to admit knowledge gaps and to refer appropriately. |
|  | Fostering professional conduct and ethics |  | Minimal levels of professionalism and ethics should be conveyed to all HCPs. |
|  | Educate on disease-specific knowledge |  | To avoid knowledge gaps, HCP should be taught disease specific knowledge. |
|  | Interpersonal skills |  | Fostering interpersonal skills in HCP helps prevent invalidation. |
|  | Communication skills | Empathetic communication | Fostering empathetic communication skills in HCP helps prevent invalidation. |
|  |  | Open communication | Fostering open communication skills in HCP helps prevent invalidation. |
|  |  | Non-verbal communication | Fostering non-verbal communication skills in HCP helps prevent invalidation. |
|  | | | |
| **Solutions on the level of patient-provider interaction** | HCPs providing clear guidance regarding future steps |  | Within the patient-provider interaction, it is essential that HCP provide clear guidance and communication about the future steps. |
|  | Shared-decision making |  | To decide these future steps, a shared-decision making approach is necessary. This can be fostered in both, HCP and patients. |
|  | Participatory communication |  | Participatory communication helps prevent invalidation. |
| **Solutions on the level of the patient** | Providing space to discuss experiences |  | Spaces should be created for patients where they can share their experiences if they have been invalidated. This can protect them from further invalidation. |
|  | Online support groups and communities |  | One of these space could be online support group or communities. They can further help patients to feel understood and supported and therefore buffer negative effects of invalidation. |
|  | Aiming for social support that accompany patients for appointments |  | Patients should be encouraged to aim for social support that accompanies them to health appointments. |
|  | Symptom tracking and documentation of HCP visits |  | Encouraging patients to track their symptoms and to document their interaction with the HCP is a prevention strategy. |
|  | Standing up for oneself |  | Helping patients to stand up for oneself can help prevent invalidation. |
|  | Searching for a doctor that takes seriously/seek second opinion |  | Patients should be encouraged – if possible from the health care structure perspective – to search for a doctor that takes them seriously or seek a second opinion. |
|  | Strategic disclosure of symptoms |  | Patients can be instructed to anticipate dismissal and to disclose symptoms strategically. |
| **Solutions for the Health Care system** | Preserving individualized health care |  | Health care should be individualized and tailored for individual patients. |
|  | Multi-disciplinary treatment options including complementary and alternative treatment options |  | Within the system, there should be multi-disciplinary treatment options, including complementary and alternative medicine. This is especially important in ambiguous, uncertain, and complex situations. |
|  | Patient-centeredness  Including a holistic and bio-psycho-social approach |  | The health care system should be patient-centered and apply a holistic and bio-psycho-social approach. |
|  | Raising awareness of implicit bias |  | Within the system it should be standard to raise implicit bias awareness. |
|  | Protection against discrimination |  | Patients should systematically protected against discrimination. |
|  | Low-treshhold health care access |  | Health care access threshold should be low, ensuring all patients have access to care. |
|  | Continuity of care |  | Within the system, continuity of care should be ensured. |
|  | Transitional health care for youth into adult care |  | Within the system, transitional health care for youth into adult care should be ensured. |
|  | | | |
| **Structural solutions** | Diversification of HCPs |  | HCP should be difersified. This includes personnel that is non-binary or BIPOC. |
|  | Fostering of narrative medicine |  | Narrative medicine should be fostered within education. |
|  | Destigmatization |  | Tools and frameworks should be identified and developed to address stigma adequately in practice and to foster destigmatization. |
|  | Combating health inequalities |  | In general, health inequalities should be combatted, as they contribute to the problem of invalidation. |
|  | Well-financed health care system | Increased specialist capacity | The health care system should be adequately funded, for example to increase specialist capacity. |
|  |  | Needed diagnostic tests are provided by insurance | The health care system should be adequately funded to ensure needed diagnostic tests are provided by insurance. |
|  |  | Sufficient time of HPC so nothing “falls under the table” | The health care system should be adequately funded to ensure the HCP has suffient time so nothing “falls under the table.” |
|  | Implementation and review of treatment and diagnostic guidelines |  | Treatment and diagnostic guidelines should regularly be reviewed and their implementation should be standard. |
|  | | | |
| **Solutions on the level of research** | Include sex/gender data in research |  | Research should include sex and gender data to ensure there is sufficient knowledge on sex-and gender specific symptoms. |
|  | More research for unexplained medical phenomena |  | Unexplained medical phenomena should be research, to have better understanding. Better understanding and knowledge can help prevent invalidation. |
