# Supplementary material for: What do we know about medical invalidation and related concepts? – A scoping review and thematic analysis about the definitions, measurements, causes, consequences and potential solutions for medical invalidation
Source: BMC Health Serv Res. 2026 Sep 8;26:1252. doi: 10.1186/s12913-026-14736-3 (PMC13560384; doi:10.1186/s12913-026-14736-3)
Supplement: Supplementary file 3 [file 12913_2026_14736_MOESM3_ESM.docx]

| **Year** | **Amount in Numbers** | **Amount in Percentage** |
| --- | --- | --- |
| 1997 | 1 | 0.63 |
| 2000 | 2 | 1.27 |
| 2001 | 1 | 0.63 |
| 2003 | 1 | 0.63 |
| 2004 | 1 | 0.63 |
| 2005 | 2 | 1.27 |
| 2006 | 2 | 1.27 |
| 2007 | 1 | 0.63 |
| 2008 | 3 | 1.90 |
| 2009 | 2 | 1.27 |
| 2010 | 1 | 0.63 |
| 2011 | 4 | 2.53 |
| 2012 | 4 | 2.53 |
| 2013 | 2 | 1.27 |
| 2014 | 4 | 2.53 |
| 2015 | 4 | 2.53 |
| 2016 | 6 | 3,80 |
| 2017 | 13 | 8.23 |
| 2018 | 4 | 2.53 |
| 2019 | 5 | 3.16 |
| 2020 | 1 | 0.63 |
| 2021 | 11 | 6.96 |
| 2022 | 25 | 15.82 |
| 2023 | 25 | 15.82 |
| 2024 | 32 | 20.25 |
| N/A | 1 | 0.63 |

**Supplement 3 – Additional descriptive tables**

Supplement Table – Year of Publication

Supplement Table – Type of Publication

| **Type of Publication** | **Amount in Numbers** | **Amount in Percentage** |
| --- | --- | --- |
| Opinion Paper / Commentary | 18 | 11.39 |
| Original Research mixed-method | 11 | 6.96 |
| Original Research qualitativ | 73 | 46.20 |
| Original Research quantitativ | 35 | 22.15 |
| Review | 19 | 12.03 |
| Meta-Synthesis of qualitative Studies | 2 | 1.27 |

Supplement Table – Country of Study Conductance

| **Country/Continent** | **Amount in Numbers** | **Amount in Percentage** |
| --- | --- | --- |
|  |  |  |
| **North America** | **60** | **37.97** |
| United States | 48 | 30.38 |
| Canada | 12 | 7.59 |
|  |  |  |
| **Europe** | **58** | **36.71** |
| Netherlands | 17 | 10.76 |
| UK | 16 | 10.13 |
| Sweden | 15 | 9.49 |
| Norway | 4 | 2.53 |
| Ireland | 2 | 1.27 |
| Belgium | 1 | 0.63 |
| Portugal | 1 | 0.63 |
| Denmark | 1 | 0.63 |
| Finnland | 1 | 0.63 |
|  |  |  |
| **Asia** | **13** | **8.23** |
| Iran | 6 | 3.80 |
| India | 3 | 1.90 |
| Israel | 1 | 0.63 |
| Pakistan | 1 | 0.63 |
| Singapore | 1 | 0.63 |
| China | 1 | 0.63 |
|  |  |  |
| **Ozeania** | **13** | **8.23** |
| Australia | 10 | 6.33 |
| New Zealand | 3 | 1.90 |
|  |  |  |
| **Southamerica** | **2** | **1.26** |
| Latin America | 1 | 0.63 |
| Chile | 1 | 0.63 |
|  |  |  |
| **International and N/A** | **12** | **7.59** |
| International (at least 2 countries) | 5 | 3.16 |
| N/A | 7 | 4.43 |

Supplement Table – Medical Specialty and Diseases

| **Medical specialty** | **Disease** | **Number** | **Percentage (%)** |
| --- | --- | --- | --- |
| Psychiatry | Total | 9 | 5.70 |
|  | Different servere mental illnesses | 6 | 3.80 |
|  | Anorexia nervosa | 1 | 0.63 |
|  | Postspartum depression | 1 | 0.63 |
|  | Premenstrual Dysphoric Disorder (PMDD) | 1 | 0.63 |
| Oncology | Total | 3 | 1.90 |
|  | Different cancers | 1 | 0.63 |
|  | Childhood cancer diagnosis | 1 | 0.63 |
|  | Ovarian cancer | 1 | 0.63 |
| Cardiology | Total | 3 | 1.90 |
|  | Different Cardiac diseases | 1 | 0.63 |
|  | Peripartum Cardiomyopathy | 2 | 1.27 |
| Gynecology | Total | 16 | 10.13 |
|  | Vulvodynia and/or duspareunia | 2 | 1.27 |
|  | PCOS | 1 | 0.63 |
|  | Heavy menstruational bleeding (HMB) | 1 | 0.63 |
|  | Endometrioses | 11 | 6.96 |
|  | Breast implant illness | 1 | 0.63 |
| Obstetrics | Total | 4 | 2.53 |
|  | Pregnant / Had giving birth | 1 | 0.63 |
|  | Severe perineal trauma (SPT) | 1 | 0.63 |
|  | Persistent pain after childbirth | 1 | 0.63 |
|  | Traumatic childbirth | 1 | 0.63 |
| Neurology | Total | 7 | 4.43 |
|  | Long Covid | 4 | 2.53 |
|  | ME/CFS | 1 | 0.63 |
|  | Long-term medically unexplained fatigue | 1 | 0.63 |
|  | ALS | 1 | 0.63 |
| Dendistry | Burning mouth syndrom (BMS) | 1 | 0.63 |
| Hematology | Hemophilia | 2 | 1.27 |
| Phlebology | Lipedema | 1 | 0.63 |
| Internal Medicine / Infectiology | Total | 4 | 2.53 |
|  | AIDS | 1 | 0.63 |
|  | Chronic lime disease | 2 | 1.27 |
|  | Post-treatment lime disease | 1 | 0.63 |
| Gastroenterology | Irritable bowel syndrom (IBS) | 1 | 0.63 |
| Rheumatology | Total | 12 | 7.59 |
|  | Different Rheumatic diseases | 5 | 3.16 |
|  | Inflammatory arthritis/ Rheumatoid arthritis | 3 | 1.90 |
|  | Lupus or realated diseases | 2 | 1.27 |
|  | Non‑inflammatory rheumatic painful disorders | 1 | 0.63 |
|  | Sjögrens Syndrome | 1 | 0.63 |
| **Disease entities** | **Specification** | **Number** | **Percentage (%)** |
| Autoimmune diseases | Different Multi-system illnesses | 1 | 0.63 |
| Multi-system illnesses | Different Multi-system illnesses | 2 | 1.27 |
| Chronic illnesses | Different Chronic illnesses | 3 | 1.90 |
| Non-cancerous pain |  | 1 | 0.63 |
| Rare diseases |  | 1 | 0.63 |
| MUPS (Medically Unexplained Physical Symptoms) |  | 6 | 3.80 |
| No concrete deasease or not applicable |  | 54 | 34.18 |
| Chronic pain | Total | 28 | 17.72 |
|  | back or neck pain | 4 | 2.53 |
|  | fibromyalgia |  |  |
|  | arthritis |  |  |
|  | headache or migraine |  |  |
|  | complex regional pain syndrome (CRPS) |  |  |
|  | „other" |  |  |
|  | cervicobrachial syndrome / fibromyalgia | 15 | 9.49 |
|  | chronic pelvic pain | 4 | 2.53 |
|  | Chronic Widespread Pain (CWP) | 1 | 0.63 |
|  | Chronic muscular pain | 1 | 0.63 |
|  | Chronic head pain | 1 | 0.63 |
|  | Chronic overlapping pain conditions (COPC) | 1 | 0.63 |
|  | Painful osteoarthritis | 1 | 0.63 |
