# Supplementary material for: What do we know about medical invalidation and related concepts? – A scoping review and thematic analysis about the definitions, measurements, causes, consequences and potential solutions for medical invalidation
Source: BMC Health Serv Res. 2026 Sep 8;26:1252. doi: 10.1186/s12913-026-14736-3 (PMC13560384; doi:10.1186/s12913-026-14736-3)
Supplement: Supplementary file 4 [file 12913_2026_14736_MOESM4_ESM.docx]

**Supplement 5 – Tables of Consequences**

*Consequences of invalidation*

| **Main Themes** | **Subthemes** | **Subsubthemes** | **Description** |
| --- | --- | --- | --- |
| **Behavioural consequences of invalidation** | Decreased Help-Seeking |  | Invalidation leads to a decreased help-seeking behaviour in patients. |
|  | Non-adherence |  | As a consequence of invalidation, patients had increased difficulties to follow treatment and guidelines to manage their symptoms. |
|  | Exaggerating symptoms to be taken seriously |  | When patients were invalidated, some started exaggerating their symptoms to achieve validation. |
|  | Self-education |  | Due to invalidation, patients increased their self-educative behaviour, e.g. through medical journals, online resources, and other sources. |
|  | | | |
| **Affective/emotional consequences of invalidation** | Feelings | Increased anger | Invalidation led to increased anger. |
|  |  | Increased frustration | Invalidation led to increased frustration. |
|  |  | Maintaining negative affect | If patients already had negative affect when visiting the HCP, this persisted after the experience of invalidation. |
|  |  | Sadness | Invalidation led to sadness. |
|  |  | Feelings of inadequacy | Invalidation led to feelings of inadequacy. |
|  |  | Feeling humiliated | Invalidation led to patients feeling humiliated. |
|  |  | Embitterment | Invalidation led to |
|  |  | Embaressment | Invalidation led to |
|  |  | Self-loathing | Invalidation led to |
|  |  | Hurt | Invalidation led to feelings of hurt. |
|  |  | Feeling like a burden | Invalidation led to feeling like a burden. |
|  |  | Shame | Invalidation led to shame. |
|  |  | Feelings of uncertainty | Invalidation led to feelings of uncertainty. |
|  |  | Reduced happiness | Invalidation led to a reduced happiness. |
|  |  | Grief | Invalidation led to grief. |
|  |  | Feeling misunderstood | Invalidation led to patients feeling misunderstood. |
|  |  | Feeling dehumanized and disrespected | Invalidation led to feeling dehumanized and disrespected. |
|  | Negative emotional consequeces | Experiencing psychological and emotional distress | Negative emotional consequences of invalidation included a general sense of emotional distress. |
|  |  | Emotional harm | Invalidation led to emotional harm. |
|  |  | Emotional dysregulation | After experiencing invalidation, some patients reported emotional dysregulation. |
|  |  | Learned helplessness | Experiencing invalidation was associated with learned helplessness. |
|  |  | Self-stigmatization | Experiencing invalidation was associated with self-stigmatization. |
|  |  | Self-invalidation | Experiencing invalidation was associated with self-invalidation. |
|  | | | |
| **Cognitive consequences of invalidation** | Increased worry |  | Invalidation led to increased worry. |
|  | Negative body-image |  | Invalidation led to a negative body-image. |
|  | Self-concept erosion |  | Invalidation erosed self-concept of patients. |
|  | Loss of self-confidence |  | Invalidation was associated with a loss of self-confidence. |
|  | Self-doubt |  | Invalidation led to self-doubt. |
|  | Deeper belief in non-scientific explanations |  | Invalidation fostered a deeper belief of patients in non-scientific explanations for their symptoms and treatment options. |
|  | Decreased health literacy |  | Invalidation led to decreased health literacy. |
|  | | | |
| **Consequences on satisfaction** | Decreased satisfaction with healthcare |  | Patients experiencing invalidation reported a decreased satisfaction with healthcare. |
|  | | | |
| **Physical health consquences** | Physical distress |  | Invalidation was associated with the experience of physical distress. |
|  | Increased symptoms including increased pain |  | Patients experiencing invalidation reported increased symptoms, often due to a lack of treatment or a missing referral to a specialist service. |
|  | Decresead life expactancy |  | Invalidation could lead to a decreased life expectancy, especially when treatment was given to late due to a missed diagnosis or no diagnosis at all. It also included wrong treatment when misdiagnosed wrongly with a mental health disorder instead of somatic disorder. |
|  | Medical complications | General Medical complications | Medical invalidation was associated with general medical complications. |
|  |  | Relapses | Medical invalidation was associated with relapses, which also could include repeated hospitalisations. |
|  |  | Close to death experiences | If a severe and acute condition was not recognized due to invalidation, this could even lead to a close to death experience. |
|  | Worsened health conditions |  | In general, experiencing of invalidation was associated with a worsened health condition. |
| **Mental health consequences** | Trauma experience | General traumatic distress | Invalidation could lead to long-term experiences of traumatic distress, including symptoms of trauma but without a full post-traumatic stress disorder. |
|  |  | Post-Traumatic Stress Syndrom | In severe cases, invalidation was a predictor of the experience of post-traumatic stress disorder. |
|  | Worsened psychological state |  | In general, long term mental health did suffer with a worsened psychological sate after experiencing invalidation. |
|  | Anxiety |  | Long term consequences of invalidation was the experience of anxiety. |
|  | Stress |  | Increased stress levels could persist in the long run after experiencing invalidation. |
|  | Depression |  | Increased stress levels could persist in the long run after experiencing depression. |
|  | Suicidal thoughts |  | Some patients who have experienced invalidation have developed suicidal thoughts. |
|  | | | |
| **Consequences for social relationships** | Social exclusion |  | Invalidation was associated with social exclusion. |
|  | Loneliness |  | Invalidation was associated with loneliness. |
|  | Isolation |  | Invalidation was associated with isolation. |
|  | Decreased support for coping with illness |  | Experiencing invalidation by a HCP could lead to decreased social support for patients. |
|  | Challenges in romantic couple relationships |  | Experiencing invalidation through a HCP could burden the romantic couple relationship, e.g. in a lack of empathy for the health condition of the patient. |
| **Consequences for health care and relationship with HPCs** | Relationship with HCP | Striving for proof | Patients started striving for proof in the relationship with the HCP. |
|  |  | Negative interaction cycles | Experiencing invalidation could lead to responses in patients, that fostered negative responses in the HCP, and so on, therefore being associated with negative interaction cycles. |
|  |  | Impaired relationship with HPC | Invalidation was associated with an impaired relationship with the HCP. |
|  | Mistrusting HPCs and loss of trust in the Health Care System |  | Experiencing invalidation fostered mistrust in both, the health care system and the HCP. |
|  | Health Services Consequences | Increased dependence on medical care | For some patients, experiencing invalidation meant an increased dependence on medical care. |
|  |  | Delayed future care-seeking or seeking care more often (doctor shopping) |  |
|  |  | Unnecessary medical interventions | After invalidation, some patients received unnecessary medical interventions. |
|  |  | Prolonged diagnostic process and delayed treatment as well as undertreatment | Invalidation could lead to prolonged diagnostic processes and delayed treatment and undertreatment. |
|  |  | Misdiagnosis | Due to invalidation, patients received increased misdiagnosis, especially mental health diagnosis instead of a correct somatic diagnosis. |
|  |  | Seeking support outside of the Health Care System |  |
|  | | | |
| **Global consequences** | Loss of trust in government |  | Experiencing invalidation fostered mistrust in the government. |
|  | Welfare denial |  | Invalidation through a HCP could also lead to welfare denial. |
|  | Higher financial costs |  | Due to multiple reasons (e.g. wrong treatment, delayed treatment, worsened condition), invalidation was associated with increased financial costs. |
|  | Employment consequences | Absentism | Experiencing invaliation affected employers, if patients then were more absent because of consequences of invalidation (e.g. worsened condition, more doctor visits). |
|  |  | Reduced work hours | Experiencing invaliation affected employers, if patients then reduced work hours because of consequences of invalidation e.g. worsened condition, more doctor visits). |

*Consequences of validation*

| Main Themes | Subthemes | Subsubthemes | Description |
| --- | --- | --- | --- |
| Behavioural consequences of validation | Being empowered |  | Patients experiencing being validated reported to be empowered to take ownership of their disease and healing process. |
|  | Adherence to treatment |  | Patients that experience validation are more likely to adhere to treatment. |
|  | | | |
| Affective/emotional consequences of validation | Feelings | Feeling humanized and respected | Validation is associated with feeling humanized and respected. |
|  |  | Feeling safe and understood | Validation is associated with feeling safe and understood. |
|  |  | Decreased anger | Validation is associated with decreased anger. |
|  |  | Decreased frustration | Validation is associated with decreased frustration. |
|  | Positive impact on affect: reduced negative affect or maintained positive affect |  | Validation had a positive effect on affect in either a reduced negative affect or maintained positive affect. |
|  | | | |
| Cognitive consequences of validation | Improved symptom comprehension (and management) |  | Validation is associated with a better understanding of ones own symptoms and how it could be managed. |
|  | Enhanced self-worth |  | Patients experiencing validation report enhanced self-worth. |
|  | Decreased worry |  | Patients experiencing validation report decreased worry. |
|  | | | |
| Consequences on satisfaction | Greater satisfcation with treatment |  | Patient experiencing validation report a greater satisfaction with treatment. |
|  | Greater life satisfaction |  | Patient experiencing validation report a greater health-related quality of life satisfactioin. |
|  | | | |
| Physical health consequences | Better treatment outcomes |  | Validation is associated with better treatment outcomes. |
|  | Decreased pain |  | Validation is associated with decreased pain. |
|  | | | |
| Consequences for health care and relationship with HPCs | Increased trust in healthcare system and HPCs |  | Patients experiencing validation reported both, increased trust in their HCP and the healthcare system. |
|  | Less feelings of entitlement to healthcare support |  | Patients experiencing validation reported a decreased sense of entitlement to healthcare support. |
|  | More likely to disclose |  | Patients experiencing validation were much more likely to engage in disclosure or relevant information towards their HCP. |
