# Supplementary material for: What do we know about medical invalidation and related concepts? – A scoping review and thematic analysis about the definitions, measurements, causes, consequences and potential solutions for medical invalidation
Source: BMC Health Serv Res. 2026 Sep 8;26:1252. doi: 10.1186/s12913-026-14736-3 (PMC13560384; doi:10.1186/s12913-026-14736-3)
Supplement: Supplementary file 5 [file 12913_2026_14736_MOESM5_ESM.docx]

**Supplement 2 – Table with Overview of Studies**

| **Author** | **Year** | **DOI** | **Country** | **Type of Study** | **Concept** | **Population** |
| --- | --- | --- | --- | --- | --- | --- |
|  |  |  |  |  |  |  |
| Abraham et al. (1) | 2024 | <https://doi.org/10.1111/jep.13974> | India | Original Research qualitative | Being disbelieved, Invalidation, (Main Focus yes/**no**) | Memoirs written by woman suffering with endometriosis |
| Agarwal (2) | 2023 | https://doi.org/10.4103/jbjd.jbjd_52_23 | India | Opinion Paper / Commentary | Gaslighting  (Main Focus **yes**/no) | N/A |
| Ahlenius et al. (3) | 2017 | http://dx.doi.org/10.1016/j.ienj.2016.07.004 | Australia | Original Research qualitative | Not being taken seriously, (Main Focus yes/**no**) | Patients (total = 32) who wrote a deviation report or complaint to the ambulance service |
| Arya et al. (4) | 2021 | https://doi.org/10.1111/jth.15311 | Canada | Original Research qualitative | Being dismissed, not being taken seriously, (Main Focus yes/**no**) | Women (total = 15) with inherited bleeding disorders, aged 18 or older |
| Au et al. (5) | 2022 | <https://doi.org/10.1016/j.ssmqr.2022.100167> | USA | Original Research mixed-method | Medical Gaslighting, (Main Focus **yes**/no) | N/A |
| Barnes (6) | 2023 | <https://doi.org/10.1093/pq/pqad035> | USA | Opinion Paper/Commentary | Gaslighting, (Main Focus **yes**/no) | N/A |
| Barry et al. (7) | 2001 | https://doi.org/10.1016/S0277-9536(00)00351-8 | UK | Original Research qualitative | Not being taken seriously, being ignored, GP-patient communication, (Main Focus yes/**no**) | Patients (total = 62 -> 35 fully analysed) visiting doctors (total = 20), aged 3 months - 80 years |
| Bidari et al. (8) | 2018 | https://doi.org/10.3344/kjp.2018.31.3.147 | Iran | Review | Invalidation, misunderstanding, mis-/underdiagnosis, (Main Focus yes/**no**) | Papers (total = 46) that describe Patients with fybromyalgia |
| Bindmann et al. (9) | 2022 | https://doi.org/10.1089/trgh.2021.0029 | USA | Original Research qualitative | Invalidation, Pathologisation, (Main Focus yes/**no**) | Nonbinary Participants (total = 24) , aged 18 or older |
| Blom et al. (10) | 2014 | Artikel hat keine DOI: PMID: 24708914 | Netherlands | Original Research quantitative | Invalidation, (Main Focus yes/**no**) | Patients who recently received the results of their disability pension examination (total = 556) and a reference group of patients without the examination (total = 96) and patients with rheumatoid arthritis (total = 32) and population reference group (total = 159) |
| Boakye et al. (11) | 2024 | <https://doi.org/10.1177/08445621241247865> | Canada | Original Research qualitative | Medical Gaslighting, medical racism, (Main Focus **yes**/no) | Black women (total = 24) who were pregnant or had given birth, aged 18 or older |
| Bönte et al. (12) | 2008 | https://doi.org/10.1016/j.whi.2008.01.003 | USA, UK, Germany | Original Research qualitative | Gender disparities, (Main Focus yes/**no**) | Primary care physicians (total = 384) |
| Bontempo (13) | 2024 | <https://doi.org/10.1177/10497323241253418> | “United States and abroad” | Original Research qualitative | Invalidation, (Main Focus **yes**/no) | Patients (total = 830) with a self-reported endometriosis diagnose, aged 18 or older |
| Bontempo (14) | 2021 (2022) | https://doi.org/10.1016/j.pec.2021.09.034 | US, Ireland, Trinidad & Tobago | N/A | Invalidation, Personalization, (Main Focus **yes**/no) | Patients (total = 609) with endometriosis |
| Bontempo (15) | 2022 | - https://doi.org/ 10.1177/13591053211024718 | USA | Review | Being dismissed or ignored, not being taken seriously, Invalidation, (Main Focus **yes**/no) | N/A |
| Boring et al. (16) | 2024 | <https://doi.org/10.1016/j.jpain.2023.11.011> | US | Original Research quantitative | Invalidation, (Main Focus **yes**/no) | Study 1: undergraduate students (total = 92)  Study 2: undergraduate students (total = 478) who reported being free of chronic pain |
| Boring et al. (17) | 2021 | <https://doi.org/10.3389/fpsyg.2021.743584> | USA | Original Research quantitative | Invalidation, (Main Focus **yes**/no) | Students (total = 478) free from current chronic pain, aged 18 or older |
| Braksmajer (18) | 2018 | <https://doi.org/10.1080/03630242.2017.1306606> | USA | Original Research qualitative | Invalidation, being dismissed, (Main Focus **yes**/no) | First phase: women with vulvodynia; second phase: also women with dyspareunia (total = 32) |
| Bransfield et al. (19) | 2019 | https://doi.org/10.3390/healthcare7040114 | US | Review | nicht ernst genommen werden  (Main Focus yes/**no**) | Clinic patients (total = 2) with Lyme borreliosis with multisystem symptoms |
| Brown et al. (20) | 2024 | https://doi.org/10.1007/s00192-023-05677-0 | USA | Original Research qualitative | Being disbelieved and dismissed, (Main Focus **yes**/no) | Female patients (total = 57), aged 19-77 |
| Bull et al. (21) | 2024 | https://doi.org/10.1177/00048674241259918 | Australia | Review | Invalidation, (Main Focus yes/**no**) | N/A (total = 16), studies that focused on patients presenting to the ED for mental health reasons |
| Cameron et al. (22) | 2018 | <https://doi.org/10.1007/s00296-017-3859-2> | International | Original Research quantitative | Invalidation, lack of understanding, being discounted, (Main Focus **yes**/no) | Participants (total = 1153) with at least one rheumatic disease and being invalidated, aged 18 or older |
| Carter (23) | 2022 | <https://doi.org/10.1002/fea2.12107> | USA | Opinion Paper/Commentary | Gaslighting, (Main Focus **yes**/no) | N/A |
| Chan et al. (24) | 2023 | https://doi.org/10.1186/s12905-023-02334-y | USA | Original Research qualitative | Medical gaslighting, (Main Focus yes/**no**) | Participants (total = 32) identifying as having PMDD, aged 18 or older |
| Chilton et al. (25) | 2021 | https://doi.org/10.1002/msc.1546 | UK | Original Research qualitative | Being dismissed or disbelieved, (Main Focus yes/**no**) | Clinic patients (total = 11) with EIA/RA |
| Christensen et al. (26) | 2022 (2023) | https://doi.org/10.1111/jocn.16511 | USA | Original Research qualitative | Being dismissed, not being taken seriously, (Main Focus yes/**no**) | Participants (total = 55) who participated in the care of a child with cancer and patients themselves if aged 16 or older |
| Christoffersen et al. (27) | 2023 | https://doi.org/10.3390/ijerph20031925 | Norway | Original Research qualitative | Not being taken seriously, (Main Focus yes/**no**) | Women (total = 15) with lipedema, aged 21-47 |
| Claassen-Van Dessel et al. (28) | 2015 | N/A | Netherlands | Original Research qualitative | To be taken seriously, (Main Focus yes/**no**) | Patients (total = 9) with at least moderate MUPS with improving symptoms |
| Cline et al. (29) | 2006 | https://doi.org/10.1016/j.socscimed.2006.03.007 | USA | Original Research qualitative | Invalidation, (Main Focus yes/**no**) | Pediatric oncology patients (total = 31), aged 3-12 years |
| Coady et al. (30) | 2024 | https://doi.org/10.1177/13591053231191919 | Canada | Original Research quantitative | Invalidation, (Main Focus **yes**/no) | Patients (total = 305) with chronic pain, aged 18 or older |
| Corry et al. (31) | 2017 | <http://dx.doi.org/10.1016/j.adolescence.2016.11.003> | UK | Original Research qualitative | Not being taken seriously(Main Focus yes/**no**) | Teenagers (total = 74) in post-primary schools, aged 13-16 |
| Côté (32) | 2024 | https://doi.org/10.1007/s11019-024-10210-1 | Canada | Review | Invalidation, epistemic injustices, (Main Focus yes/**no**) | Articles (total = 35) about epistemic justice |
| Davis (33) | 2022 | https://medcraveonline.com/MOJAMT/MOJAMT-07-00151.pdf | USA | Opinion Paper/ Commentary | Gaslighting, (Main Focus **yes**/no) | N/A |
| De León-Menjivar (34) | 2022 | <https://doi.org/10.1177/15404153211064608> | US | Original Research qualitative | Being dismissed, medical gaslighting, (Main Focus yes/**no**) | Women (total = 6) with fibromyalgia |
| Dekker et al. (35) | 2016 | <https://doi:10.1111/jmwh.12448> | USA | Original Research qualitative | Being dismissed, not being taken seriously, (Main Focus yes/**no**) | Women (total = 92) with PPCM in online support groups |
| DeRuddere et al. (36) | 2013 | http://dx.doi.org/10.1016/j.pain.2012.12.018 | Belgium | Original Research qualitative | Being discounted, not taking seriously, (Main Focus **yes**/no) | Participants (total = 62), aged 18 or older |
| Dieppe et al. (37) | 2016 | http://dx.doi.org/10.1016/j.joca.2016.06.007 | UK | Review | Validation, Invalidation, (Main Focus yes/**no**) | N/A |
| Durbhakula et al. (38) | 2023 | <https://doi.org/10.1007/s11606-023-08302-4> | USA | Opinion Paper/ Commentary | Gaslighting, (Main Focus **yes**/no) | N/A |
| Eder et al. (39) | 2024 (2023) | <https://doi.org/10.1080/26895269.2023.2286268> | Canada, Norway, Australia, USA, New Zealand, Ireland | Original Research qualitative | Medical gaslighting, (Main Focus yes/**no**) | Participants (total = 11) who identify as transgender and/or non-binary with suspected endometriosis, aged 18 or older |
| Edlund et al. (40) | 2017 | https://doi.org/10.1016/j.sjpain.2017.07.007 | Sweden | Original Research quantitative | Validation and Invalidation  (Main Focus **yes**/no) | Swedish pain patients (total 395) with age 21-62 (Ø 45.50 years) |
| Edlund et al. (41) | 2023 | https://doi.org/10.1016/j.ejon.2023.102401 | Sweden | Original Research quantitative | Validation, Invalidation, (Main Focus yes/**no**) | Contact nurses (total = 18) working in cancer or palliative care |
| Edmond and Keefe (42) | 2015 | https://doi.org/10.1097/01.j.pain.0000460301.18207.c2 | USA | Review | Validation, Invalidation, (Main Focus **yes**/no) | N/A |
| Ellis et al. (43) | 2023 | 10.3389/fmed.2023.1185769 | New Zealand | Original Research mixed-method | (Main Focus **yes**/no) | Participants (total = 50) with endometriosis, aged 18-48 |
| Eriksen et al. (44) | 2023 | https://doi.org/10.1111/jocn.16704 | Norway (including studies from other countries) | Qualitativee Metasythesis | Not being taken seriously, (Main Focus yes/**no**) | Peer-reviewed primary qualitativee studies (total = 24) on negative patient experience (formal complaints) |
| Fagen et al. (45) | 2024 | <https://doi.org/10.3390/healthcare12010078> | 28 different countries, the  majority (95%) residing in United States, Australia, Canada, Ireland, UK | Original Research quantitative | Gaslighting, (Main Focus **yes**/no) | Patients with Lyme disease/CLD or having minor children with Lyme disease/CLD (total = 986), patients aged 18 or older |
| Ferguson et al. (46) | 2019 | https://doi.org/10.1111/hex.12886 | Australia | Original Research qualitative | Communication problems, lack of professionalism and compassion, (Main Focus yes/**no**) | Males (total = 30) who accessed ambulance service for mental health or AOD problem, aged 18 or older |
| Fielding-Singh et al. (47) | 2022 | https://doi.org/10.1016/j.socscimed.2022.114938 | USA | Original Research qualitativee | (obstetric) Gaslighting, (Main Focus **yes**/no) | Participants (total = 46) who gave birth (difficult, traumatic) to a living child two or more months prior |
| Frostholm et al. (48) | 2005 | http://doi.org/10.1097/01.psy.0000188403.94327.5b | Denmark | Original Research quantitative | Invalidation  (Main Focus yes/**no**) | Patients (total = 1785) with functional disorder and somatization with age 18-65 |
| Garcia (49) | 2024 | https://doi.org/10.1016/j.ijnurstu.2024.104911 | USA | Qualitativee content analysis | Obstretic violence, mistreatment, disrespect, abuse, (Main Focus yes/**no**) | Childbearing/birthing people and people caring for them (total = 139, 125 of them birthing) |
| Geenen (50) | 2009 | https://doi.org/10.1080/10582450802675951 | Netherlands | Opinion Paper/Commentary | Disbelief, stigma, lack of acceptance, (Main Focus yes/**no**) | N/A (in March 1998 6.000 attended 50 meetings and 300 physicians/health professionals attended a conference) |
| Gertsman et al. (51) | 2023 | https://doi.org/10.9778/cmajo.20220211 | USA | Original Research qualitative | Invalidation, (Main Focus yes/**no**) | Patients (total = 20) with chronic illness, aged 18 or older |
| Ghavidel-Parsa et al. (52) | 2021 | <https://doi.org/10.1007/s10067-020-05515-4> | Iran | Original Research quantitative | Invalidation, discounting, lack of understanding, (Main Focus **yes**/no) | Female patients (total = 165) with a new Fibromyalgia (FM) diagnosis and patients (total = 62) with chronic painful non-inflammatory conditions (non-FM), aged 18-65 |
| Ghavidel-Parsa et al. (8) | 2015 | <https://doi.org/10.1093/rheumatology/keu355> | Iran | Original Research quantitative | Invalidation, discounting, lack of understanding, (Main Focus **yes**/no) | Female patients (total = 112) with fibromyalgia, aged 18-61) |
| Ghavidel-Parsa et al. (53) | 2022 (2021) | https://doi.org/10.1007/s10067-021-05890-6 | Iran | Original Research quantitative | Social Pain (Invalidation), (Main Focus **yes**/no) | Female patients (total = 81) with fibromyalgia, aged 18-65 |
| Ghavidel-Parsa et al. (53) | 2022 | <https://doi.org/10.1186/s41927-022-00256-0> | Iran | Original Research quantitative | Invalidation, (Main Focus yes/**no**) | Patients (total = 196) with chronic non-inflammatory musculoskeletal pain |
| Gillie et al. (54) | 2024 | <https://doi.org/10.1111/nin.12669> | Israel | Opinion Paper/Commentary | Gaslighting, (Main Focus **yes**/no) | Patients in medical system |
| Godman (55) | 2024 | <https://www.health.harvard.edu/staying-healthy/what-to-do-about-medical-gaslighting> | USA | Opinion Paper/Commentary | Gaslighting, (Main Focus **yes**/no) | N/A |
| Goff et al. (56) | 2000 | https://doi.org/10.1002/1097-0142(20001115)89:10%3C2068::AID-CNCR6%3E3.0.CO;2-Z | USA | Journal article | Being ignored, (Main Focus yes/**no**) | Surveys answered by female patients (total = 1725) with ovarian cancer |
| Greville-Harris (37) | 2017 | http://dx.doi.org/10.1016/j.sjpain.2017.07.007. | UK | Opinion Paper/ Commentary | Validation, Invalidation, (Main Focus **yes**/no) | Patients having a multimodal pain rehabilitation |
| Greville-Harris et al. (57) | 2015 | <http://dx.doi.org/10.1016/j.amjmed.2014.08.031> | UK | Review | Validation, Invalidation, Nocebo, (Main Focus **yes**/no) | N/A |
| Grundström et al. (58) | 2017 | <https://doi.org/10.1111/jocn.13872> | Sweden | Qualitativee study original | Being treated with ignorance, being acknowledged, (Main Focus yes/**no**) | Women (total = 12) with endometriosis, aged 18 or older |
| Grundström et al. (59) | 2023 | https://doi.org/10.1080/0167482X.2023.2264483 | Sweden | Journal Article | Invalidation, Validation, being disqualified, (Main Focus **yes**/no) | Biological women (total = 427) with self-reported endometriosis, aged 18 or older |
| Gunning (60) | 2023 | https://doi.org/10.1177/02654075221137548 | USA | Original Research qualitative | Being discredited and dismissed, Invalidation, (Main Focus **yes**/no) | Assigned female at birth (total = 25: 24 cisgender women, 1 male AFAB) diagnosed with an autoimmune disease, aged 19-30 |
| Haraldsson et al. (61) | 2024 | <https://doi.org/10.1186/s12875-024-02431-3> | Sweden | Original Research qualitative | Being ignored, dismissed or belittled, (Main Focus yes/**no**) | Adolescent males (total = 9) who visited any of the GPs included in the project, aged 15-19 |
| Hausmann et al. (62) | 2018 | https://doi.org/10.1186/s13023-018-0902-7 | USA | Original Research qualitative | Invalidation  (Main Focus yes/**no**) | Patients (total = 14) with autoinflammatory diseases with age 1-28 |
| Hearn et al. (63) | 2020 | https://doi.org/10.1016/S2468-1253(19)30348-6 | UK | Review | Not being taken seriously, (Main Focus yes/**no**) | Existing research and studies (total = 20) on irritable bowel syndrome |
| Henry et al. (64) | 2023 | <https://doi.org/10.1002/ijgo.14945> | New Zealand | Supplement Article | Medical gaslighting, Normalization, feeling unheard, self-Invalidation, (Main Focus **yes**/no) | Research reports and studies (total = 13) describing women’s experiences of heavy bleeding during their periods |
| Herrera et al. (65) | 2024 | https://doi.org/10.3390/jcm13113228 | Chile | Original Research qualitative | Invalidation, (Main Focus yes/**no**) | Women (total = 15) with a confirmed Sjögrens Syndrome, aged 26-68 |
| Hildebrand et al. (66) | 2022 | https://doi.org/ps://doi.org/10.1177/15248399211027540 | USA | Original Research quantitative | Being dismissed, not being taken seriously (Main Focus **yes**/no) | Patients (total = 763) who report being dismissed by medical professionals |
| Hintz (67) | 2023 | https://doi.org/10.1080/10410236.2022.2081046 | USA | Meta-Synthesis of a Qualitativee Research | Being dismissed, Invalidation, “it’s all in your head”, (Main Focus yes/**no**) | Qualitativee interview studies (total = 82) on patient-provider communication, female patients (total = 2.434) with chronic overlapping pain conditions |
| Holopainen et al. (68) | 2021 (2023) | https://doi.org/10.1080/21679169.2021.1967446 | Finnland | Original Research mixed-method | Validation, Invalidation, (Main Focus **yes**/no) | Physiotherapists (total = 18) who received CFT trainings |
| Houwen et al. (69) | 2017 | <https://doi.org/10.1093/fampra/cmw132> | Netherlands | Original Research qualitative | Being taken seriously, (Main Focus **yes**/no) | Patients with medically unexplained symptoms ad general practitioners (total = 393), aged 18 or older |
| Houwen et al. (70) | 2017 | https://doi.org/10.3399/bjgp17X692537 | Netherlands | Original Research qualitative | Not being taken seriously, (Main Focus **yes**/no) | Patients (total = 393 -> n = 43 MUS patients) with medically unexplained symptoms |
| Houwen et al. (70) | 2017 (2016) | <https://doi.org/10.1093/fampra/cmw132> | Netherlands | Original Research qualitative | Being taken seriously, (Main Focus yes/**no**) | Patients (total = 39) with medically unexplained symptoms, aged 18 or older |
| Hutchens et al. (71) | 2023 | https://doi.org/10.1080/17482631.2023.2182953 | Australia | Original Research qualitative | Invalidation, being dismissed, (Main Focus yes/**no**) | Women (total = 25) who had cardiac disease during pregnancy |
| Jannesari (72) | 2024 | N/A | USA | Doctoral Dissertation | Medical Gaslighting  (Main Focus yes/**no**) | 225 Non-BIPOC and 86 BIPOC (total 311) with Endometriosis with age 18-65 |
| Järemo (73) | 2014 | https://doi.org/10.1186/s40359-014-0057-9 | Sweden | Original Research mixed-methods | Invalidation (Ilness Invalidation Inventory) (Main Focus **yes**/no) | Swedish patients (total = 11) with chronic pain, fibromyalgia, Cervicobrachial Syndrome |
| Järemo et al. (73) | 2022 | <https://doi.org/10.2340/jrm.v54.67> | Sweden | Original Research quantitative | Invalidation, (Main Focus **yes**/no) | Patients (total = 152) with chronic widespread pain, aged 18 or older |
| Jedick (74) | 2022 | <https://doi.org/10.1097/01.EEM.0000891156.61054.4a> | USA | Opinion Paper/Commentary | Gaslighting, being dismissed, (Main Focus **yes**/no) | N/A |
| Katz et al. (75) | 2000 | N/A | USA | Original Research qualitative | Being taken seriously, (Main Focus yes/**no**) | N/A |
| Kauppi et al. (76) | 2023 | https://doi.org/10.1111/scs.13108 | Sweden | Original Research qualitative | Not being taken seriously, (Main Focus **yes**/no) | Patients (total = 14) who experienced breathlessness and were cared for by ambulance clinicians, aged 18 or older |
| Kendrick et al. (77) | 2016 | http://dx.doi.org/10.1080/21641846.2016.1250862 | USA | Original Research qualitative | Invalidation, (Main Focus **yes**/no) | Patients (total = 187) with CFS, aged 19-78 |
| Khan et al. (78) | 2024 | https://doi.org/10.52053/jpap.v5i1.249 | Pakistan | Review | Medical gaslighting, (Main Focus **yes**/no) | Original research articles (total = 10) about experiences of medical gaslighting in women |
| Kieren et al. (79) | 2023 | https://doi.org/10.1016/j.acap.2023.06.008 | USA | Original Research qualitative | Being dismissed or ignored, Validation, Invalidation, (Main Focus yes/**no**) | Parents (total = 31) who had a hospitalized child |
| King et al. (80) | 2024 | https://doi.org/10.1080/17522439.2022.2141841 | UK | Original Research qualitative | Not being believed or taken seriously, (Main Focus yes/**no**) | Participants (total = 9) who had taken antipsychotic drugs for any indication, aged 18 or older |
| Kool et al. (81) | 2010 | <https://doi.org/10.1136/ard.2009.123224> | Netherlands | Original Research quantitative | Invalidation, being discounted, (Main Focus **yes**/no) | Patients (total = 319) with rheumatic diseases and experiences with Invalidation, aged 18 or older |
| Kool et al. (82) | 2012 (2013) | <https://doi.org/10.1177/1359105312436438> | Netherlands | Original Research qualitative | Invalidation, social support, (Main Focus **yes**/no) | Participants (total = 1455) with a rheumatic disease |
| Kool et al. (83) | 2012 | <https://doi.org/10.1080/00223980.2011.606434> | Netherlands | Original Study quantitative | Invalidation, (Main Focus **yes**/no) | Patients (total = 927) with ankylosing spondylitis, fibromyalgia, osteoarthritis, rheumatoid arthritis or systemic diseases, aged 18 or older |
| Kool et al. (84) | 2012 | https://doi.org/10.1080/00223980.2011.606434 | Netherlands | Original Research quantitative | Invalidation, (Main Focus yes/**no**) | Patients (total = 927) with rheumatic diseases, aged 18 or older |
| Kool et al. (84) | 2011 | N/A | Netherlands | Original Research quantitative | Invalidation, being discounted, (Main Focus **yes**/no) | Patients and spouses (total = 197) with either rheumatoid arthritis or fibromyalgia |
| Kool et al. (85) | 2012 (2014) | https://doi.org/10.1136/annrheumdis-2012-201807 | Netherlands | Original Research quantitative | Invalidation, (Main Focus yes/**no**) | Participants (total = 6057) with rheumatic diseases, aged 18 or older |
| Laestadius et al. (86) | 2024 | https://doi.org/10.1186/s12916-024-03465-1 | United States | Original Research qualitative | (lack of) Validation, (Main Focus yes/**no**) | Women (total = 30) who experienced Long COVID symptoms, aged 18 or older |
| Larrea et al. (87) | 2021 | <https://doi.org/10.1080/10130950.2021.1975967> | Latin America | Original Research qualitative | Gaslighting, Obstetric violence, (Main Focus yes/**no**) | Women (total = 20) who reported being subjected to several forms of obstetric violence |
| Lesnewich et al. (88) | 2023 | https://doi.org/10.1080/08870446.2023.2248481 | USA | Original Research qualitative | Validation, Invalidation, (Main Focus yes/**no**) | Gulf War Veterans (total = 31) with different chronic symptoms |
| Lian et al. (89) | 2017 | <https://doi.org/10.1080/17482631.2017.1392219> | Norway | Original Research qualitative | Medical uncertainty, doctor-patient interactions, (Main Focus yes/**no**) | Participants (total = 256) who responded to “Is  there something that you want to tell us that you have not already done so far in this survey?”” |
| Liggins et al. (90) | 2005 | https://doi.org/10.1016/j.genhosppsych.2005.05.006 | New Zealand | Original Research qualitative | Invalidation, (Main Focus **yes**/no) | Clinic Patients and health professionals (total = 10), patients aged 18-65 |
| Lightbourne et al. (91) | 2024 | https://doi.org/10.1177/10497323231214114 | Ireland | Original Research qualitativee | Being dismissed, (Main Focus yes/**no**) | Participants (total = 20) with endometriosis, aged 18- late 50s |
| Linton et al. (92) | 2017 | https://doi.org/10.1097/PR9.0000000000000600 | Sweden | Original Research quantitative | Validation, Invalidation (Main Focus **yes**/no) | University Students (total = 22) |
| Lobo et al. (93) | 2014 | https://doi.org/10.4088/PCC.14m01664 | USA | Original Research quantitative | Invalidation, Validation, (Main Focus **yes**/no) | Patients (total = 670) with Fybromyalgia, aged 18 or older |
| López-Aybar et al. (94) | 2024 (2023) | https://doi.org/10.1037/ser0000765 | USA | Original Research mixed-method | Invalidation, Stigma, (Main Focus yes/**no**) | Prosumers (total = 175) with any DSM-5 diagnosis, aged 18 or older |
| Lund et al. (95) | 2024 | https://doi.org/10.1097/AJP.0000000000001238 | Canada | Original Research qualitative | Invalidation, (Main Focus yes/**no**) | Parent-child dyads (total = 26) where the child has chronic head pain, aged 11-18 |
| Mathur et al. (96) | 2023 | https://doi.org/10.1093/pm/pnad078 | USA | Original Research quantitative | Stigma, discrimination, Invalidation, (Main Focus yes/**no**) | Women (total = 16) with burning mouth syndrome, aged 40-85 |
| Molin et al. (97) | 2024 | https://doi.org/10.1016/j.srhc.2023.100929 | Sweden | Original Research qualitativee | Invalidation, not being taken seriously (Main Focus yes/**no**) | Women (total = 20) with persisting pain after childbirth |
| Mullard et al. (98) | 2024 | https://doi.org/10.1111/1467-9566.13795 | UK | Original Research qualitative | Validation, Invalidation, (Main Focus yes/**no**) | Health or community professionals working in the field of COVID-19 or Long Covid (total = 18) and Long Covid-patients (total = 23) |
| Munro et al. (99) | 2022 | https://doi.org/10.1080/08870446.2021.1912344 | USA | Original Research qualitative | Invalidation, lack of understanding, (Main Focus yes/**no**) | Patients (total = 384) with rare disease(s), aged 18 or older |
| Newsom et al. (100) | 2022 | https://doi.org/10.1089/trgh.2021.0027 | USA | Original Research mixed-method | Invalidation, (Main Focus yes/**no**) | Participants (total = 85) not identifying as cisgender, aged 14 or older |
| Ng et al. (101) | 2024 | <https://doi.org/10.1016/j.amjmed.2024.06.022> | Singapore | Opinion Paper/Commentary | Gaslighting, Invalidation, (Main Focus **yes**/no) | N/A |
| Nicola et al. (102) | 2022 | https://10.3389/fpain.2022.884335 | Australia | Review | Pain-Validation and Pain-Invalidation, (Main Focus **yes**/no) | N/A |
| Nicola et al. (103) | 2022 | https://doi.org/10.1016/j.jpain.2022.06.008 | United States | Original Research mixed-method | Invalidation (Main Focus **yes**/no) | Patients (total = 910) with chronic pain, aged 18-65 |
| Nicola et al. (104) | 2019 (2021) | https://doi.org/10.1080/09638288.2019.1636888 | Australia | Review | Invalidation, Validation, (Main Focus **yes**/no) | Peer-reviewed journal articles (total = 431) containing participants (total over 7770) with pain conditions, aged 18-65 |
| Niedenfuehr et al. (105) | 2023 | https://doi.org/10.1093/jsxmed/qdad035 | USA | Review | Invalidation, (Main Focus yes/**no**) | Articles (total = 73) that focus on vulvodynia |
| Nishikawara et al. (106) | 2023 | https://doi.org/10.1080/24740527.2023.2176745 | Canada | Original Research qualitative | Invalidation, (Main Focus yes/**no**) | Patients (total = 14) with fibromyalgia, aged 18 or older |
| O’Malley et al. (107) | 2019 (2020) | https://doi.org/10.1111/jppi.12323 | Ireland | Original Research qualitative | Not being taken seriously, Invalidation, (Main Focus yes/**no**) | Clinical psychologists (total = 6) working in adult intellectual disability services |
| Oosterhof et al. (108) | 2014 | <https://doi.org/10.3109/09638288.2014.881566> | Netherlands | Original Research qualitative | (Main Focus yes/**no**) | Patients (total = 16) with chronic pain and healthcare professionals (total = 10) |
| Owen et al. (109) | 2024 | <https://doi.org/10.1007/s11136-023-03513-y> | UK | Opinion Paper/ Commentary | Gaslighting, (Main Focus **yes**/no) | 132 patients diagnosed with Long Covid |
| Patel et al. (110) | 2016 | <https://doi.org/10.1186/s12884-016-1178-3> | Sweden | Original Research qualitative | Not being taken seriously, not being cared about, (Main Focus yes/**no**) | Women (total = 19) with PPCM (Peripartum Cardiomyopathy) |
| Plackowski et al. (111) | 2023 | https://doi.org/10.1177/10497323221135974 | USA | Original Research qualitative | Invalidation, being dismissed, gaslighting, (Main Focus yes/**no**) | Patients (total = 27) with rare disorders, aged 18 or older |
| Price et al. (112) | 2006 | <https://doi.org/10.1111/j.1471-0528.2006.00862.x> | UK | Original Research qualitativee | Being taken seriously, (Main Focus **yes**/no) | Women (total = 26) attending a clinic and having CPP (chronic pelvic pain), aged 18-70 |
| Qureshi et al. (113) | 2011 (2021) | https://doi.org/10.7759/cureus.18692 | China | Review | Diagnostic Challenges, Invalidation, disbelief, (Main Focus yes/**no**) | N/A |
| Rebman et al. (114) | 2024 | <https://doi.org/10.1038/s41598-024-70556-7> | USA | Original Research quantitative | Invalidation, (Main Focus **yes**/no) | Patients (total = 99) who met criteria for PTLD |
| Renault et al. (115) | 2011 | <https://doi.org/org/10.1111/j.1365-2516.2010.02424.x> | Canada | Original Research qualitative | Not being believed, being dismissed, (Main Focus yes/**no**) | Haemophilia carriers (total = 11) or their mothers, aged 18 or older |
| Riggs et al. (116) | 2018 | https://doi.org/10.1080/14681994.2018.1444274 | Australia | Opinion Paper / Commentary | Gaslighting (Main Focus **yes**/no) | N/A |
| Russel et al. (117) | 2022 | https://doi.org/10.1016/j.ssmqr.2022.100177 | USA | Original Research qualitative | Validation, Invalidation, gaslighting, (Main Focus **yes**/no) | Patients (total = 27) with Long COVID, aged 18 or older |
| Sakalys (118) | 1997 | https://doi.org/10.1002/art.1790100404 | USA | Original Research mixed-method | Invalidation, Disconfirmation, Normalization, (Main Focus **yes**/no) | Female patients (total = 50) with Rheumatoid Arthritis, aged 20-60 |
| Salmon (119) | 2007 | <https://doi.org/10.1016/j.pec.2007.03.008> | UK | Review | Not being taken seriously, (Main Focus yes/**no**) | Papers with patients with medical unexplained symptoms |
| Samsson et al. (120) | 2017 | <https://doi.org/10.1186/s12891-017-1719-6> | Sweden | Original Research qualitative | Being taken seriously, (Main Focus **yes**/no) | Patients (total = 13) who were referred for an orthopaedic consultation, aged 18-67 |
| Samulowitz (121) | 2018 | https://doi.org/10.1155/2018/6358624 | Sweden | Review | Not being taken seriously (Main Focus yes/**no**) | Articles (total = 77) describing chronic pain and gender norms |
| Santiago et al. (122) | 2017 | <https://doi.org/10.3899/jrheum.160559> | Portugal | Original Research quantitative | Invalidation, (Main Focus **yes**/no) | Participants (total = 746) with a rheumatic disease, aged 18 or older |
| Schäfer et al. (123) | 2016 | https://doi.org/10.1097/j.pain.0000000000000536 | UK | Original Research quantitative | Not being taken seriously, (Main Focus yes/**no**) | Doctors working in pain services, medical students (4^th^-5^th^ year) |
| Sebring (124) | 2021 | <https://doi.org/10.1111/1467-9566.13367> | USA | Review | Gaslighting, (Main Focus **yes**/no) | N/A |
| Sebring et al. (125) | 2023 | N/A | Canada | Original Research qualitative | Invalidation, being dismissed, (Main Focus **yes**/no) | Women and non-binary people (total = 10) with chronic illness |
| Sells et al. (126) | 2008 | N/A | US | Original Research quantitative | Invalidation, Validation, (Main Focus **yes**/no) | Patients (total = 137) with severe mental disorder |
| Sharpe (127) | 2022 | <http://dx.doi.org/10.1136/bmj.o2236> | UK | Mixed-Method Online Survey | Gaslighting, (Main Focus **yes**/no) | N/A |
| Sheikhan et al. (128) | 2023 | https://doi.org/10.1186/s12913-023-09075-6 | Canada | Original Research qualitative | Not being believed, being minimized or dismissed, (Main Focus yes/**no**) | Youth participants (total = 22) with lived experiences of mental health, aged 14-25 |
| Singh et al. (129) | 2021 | <https://doi.org/10.1111/1756-185X.14163> | India | Original Research quantitative | Invalidation, (Main Focus **yes**/no) | Patients (total = 157) with rheumatic arthritis or fibromyalgia, aged 18 or older |
| Sloan et al. (130) | 2021 | <https://doi.org/10.1007/s00296-020-04726-x> | UK | N/A | Invalidation, (Main Focus **yes**/no) | Forum posts of participants (total 2649) with Lupus |
| Sloan et al. (131) | 2020 | https://doi.org/10.1093/rap/rkaa006 | UK | Original Research mixed-methods | Invalidation  (Main Focus yes/**no**) | Patients (total 233) with lupus, UCTD, MCTD, SS or overlap condition with age 18 |
| Soucie et al. (132) | 2022 (2023) | https://doi.org/10.1080/23293691.2022.2054671 | Canada | Original Research qualitative | Being dismissed and discredited, “brushed off”, “ignored”, “not taken seriously” | Woman (total = 72) with Polycystic Ovary Syndrome |
| Staal et al. (133) | 2016 | https://doi.org/10.1159/000441911 | Netherlands | Original Research quantitative | Delayed/Mis- diagnosis, (Main Focus yes/**no**) | Women (total = 93) with endometriosis, aged 18 or older |
| Stone (82) | 2013 | N/A | Australia | Original Research qualitative | Validation, (Main Focus yes/**no**) | GP’s (total = 24) working with patients with medical unexplained symptoms |
| Sword et al. (134) | 2008 | <https://doi.org/10.1177/1049732308321736> | Canada | Original Research qualitative | Normalizing, (Main Focus yes/**no**) | Women (total = 18) with positive screening for postpartum depression |
| Tang et al. (135) | 2022 | https://doi.org/10.1093/asj/sjab204 | Australia | Original Research qualitative | Invalidation, (Main Focus yes/**no**) | Woman (total = 29) with breast implant illness |
| Thomas’s (136) | 2024 | https://doi.org/10.1038/d41586-024-03005-0 | N/A | Opinion Paper / Commentary | Being dismissed or ignored,  (Main Focus yes/**no**) | N/A |
| Thompson et al. (137) | 2023 | https://doi.org/10.1080/10410236.2022.2137772 | USA | Original Research qualitative | Not being taken seriously  (Main Focus **yes**/no) | Women (total = 36) with Endometriosis |
| Thompson et al. (138) | 2025 | https://doi.org/10.1177/00936502231184318 | USA | Original Research qualitative | Being dismissed, Not being taken seriously, Gaslighting, Invalidation  (Main Focus **yes**/no) | Women (total = 36) |
| Tjernström (139) | 2024 | https://doi.org/10.1186/s12913-024-11037-5 | Sweden | Original Research qualitative | Obstretic Gaslighting, Invalidation / Not being taken seriously  (Main Focus **yes**/no) | Women (total = 12) with severe perineal trauma (SPT) with age above 18 years |
| Treufeldt et al. (140) | 2024 | https://doi.org/10.1016/j.pec.2024.108198 | UK | Review | Stigma, Invalidation  (Main Focus **yes**/no) | Total = 32 papers |
| Van Middendorp et al. (141) | 2009 | <https://doi.org/10.1002/art.24922> | Netherlands | Original Research qualitative | Invalidation, (Main Focus **yes**/no) | Participants (total = 10) with fibromyalgia, aged 18 or older |
| Vangronsveld et al. (142) | 2011 (2012) | https//doi.org/10.1016/j.ejpain.2011.07.009 | Sweden | Original Research mixed-method | Validation, Invalidation, (Main Focus **yes**/no) | Nurses (total = 28) with back and musculoskeletal pain |
| Verbeek et al. (143) | 2004 (2005) | https://doi.org/ 10.1136/oem.2004.016303 | Netherlands | Original Research quantitative | Being taken seriously, (Main Focus yes/**no**) | Patients (total = 432) who have been recently seen by an occupational health physician |
| Vriezekolk et al. (144) | 2019 | <https://doi.org/10.1093/rap/rkz008> | Netherlands | Original Research quantitative | Invalidation  (Main Focus **yes**/no) | Clinic patients (total = 280) with fibromyalgia with age 18 or older |
| Walkden (145) | 2024 (2023) | <https://doi.org/10.1080/10410236.2023.2296189> | N/A | Opinion Paper/Commentary | Gaslighting, (Main Focus **yes**/no) | N/A |
| Wasson Simpson et al. (146) | 2022 | https://doi.org/10.1007/s10488-021-01177-9 | Canada | Original Research qualitative | Validation, Invalidation, not being taken seriously, (Main Focus **yes**/no) | Youth (total = 31) with mental health disorder, diagnosed or self-identified |
| Werner et al. (147) | 2003 | <https://doi.org/10.1016/S0277-9536(02)00520-8> | Norway | N/A | Validation, Invalidation, (not) being taken seriously, (Main Focus **yes**/no) | Women (total = 10) with chronic muscular pain |
| Wise (148) | 2022 | <http://dx.doi.org/10.1136/bmj.o1974> | N/A | Opinion Paper/Commentary | Gaslighting, (Main Focus **yes**/no) | N/A |
| Yadava (149) | 2023 | <https://doi.org/10.1007/s12055-023-01588-3> | N/A | Opinion Paper/Commentary | Gaslighting, forced demand for altruism, (Main Focus **yes**/no) | N/A |
| Zugai et al. (150) | 2024 | https://doi.org/10.1111/inm.13242 | Australia | Original Research qualitative | Invalidation, (Main Focus yes/**no**) | Participants (total = 10) who had previously been hospitalised for the treatment of anorexia nervosa, aged 18 or older |

**Literature**

1.	Abraham JM, V. R. Emotions of endometriosis in clinical encounters: An analysis of women’s experiences of health care. J Eval Clin Pract. Juni 2024;30(4):575–81.

2.	Agarwal A. Are we ready to accept the existence of medical gaslighting? An urgent call for awareness and advocacy. J Bone Jt Dis. September 2023;38(3):217–8.

3.	Ahlenius M, Lindström V, Vicente V. Patients’ experience of being badly treated in the ambulance service: A qualitative study of deviation reports in Sweden. Int Emerg Nurs. Januar 2017;30:25–30.

4.	Arya S, Wilton P, Page D, Boma‐Fischer L, Floros G, Winikoff R, u. a. “They don’t really take my bleeds seriously”: Barriers to care for women with inherited bleeding disorders. J Thromb Haemost. Juni 2021;19(6):1506–14.

5.	Au L, Capotescu C, Eyal G, Finestone G. Long covid and medical gaslighting: Dismissal, delayed diagnosis, and deferred treatment. SSM - Qual Res Health. Dezember 2022;2:100167.

6.	Barnes E. Trust, Distrust, and ‘Medical Gaslighting’. Philos Q. 12. Juni 2023;73(3):649–76.

7.	Barry CA, Stevenson FA, Britten N, Barber N, Bradley CP. Giving voice to the lifeworld. More humane, more effective medical care? A qualitative study of doctor–patient communication in general practice. Soc Sci Med. August 2001;53(4):487–505.

8.	Bidari A, Parsa BG, Ghalehbaghi B. Challenges in fibromyalgia diagnosis: from meaning of symptoms to fibromyalgia labeling. Korean J Pain. 31. Juli 2018;31(3):147–54.

9.	Bindman J, Ngo A, Zamudio-Haas S, Sevelius J. Health Care Experiences of Patients with Nonbinary Gender Identities. Transgender Health. 1. Oktober 2022;7(5):423–9.

10.	Blom E, Küntay AC, Messer M, Verhagen J, Leseman P. The benefits of being bilingual: Working memory in bilingual Turkish–Dutch children. J Exp Child Psychol. Dezember 2014;128:105–19.

11.	Boakye PN, Prendergast N, Bailey A, Sharon M, Bandari B, Odutayo A ama, u. a. Anti-Black Medical Gaslighting in Healthcare: Experiences of Black Women in Canada. Can J Nurs Res. März 2025;57(1):59–68.

12.	Bönte M, Von Dem Knesebeck O, Siegrist J, Marceau L, Link C, Arber S, u. a. Women and Men with Coronary Heart Disease in Three Countries: Are They Treated Differently? Womens Health Issues. Mai 2008;18(3):191–8.

13.	Bontempo AC. Conceptualizing Symptom Invalidation as Experienced by Patients With Endometriosis. Qual Health Res. Februar 2025;35(2):248–63.

14.	Bontempo AC. The effect of personalized invalidation of symptoms by healthcare providers on patient depression: The mediating role of self-esteem. Patient Educ Couns. Juni 2022;105(6):1598–605.

15.	Bontempo AC. The need for a standardized conceptual term to describe invalidation of patient symptoms. J Health Psychol. August 2022;27(9):2104–14.

16.	Boring BL, Walsh KT, Ng BW, Schlegel RJ, Mathur VA. Experiencing Pain Invalidation is Associated with Under-Reporting of Pain: A Social Psychological Perspective on Acute Pain Communication. J Pain. Mai 2024;25(5):104428.

17.	Boring BL, Walsh KT, Nanavaty N, Mathur VA. Shame Mediates the Relationship Between Pain Invalidation and Depression. Front Psychol. 3. Dezember 2021;12:743584.

18.	Braksmajer A. Struggles for medical legitimacy among women experiencing sexual pain: A qualitative study. Women Health. 21. April 2018;58(4):419–33.

19.	Bransfield RC, Friedman KJ. Differentiating Psychosomatic, Somatopsychic, Multisystem Illnesses and Medical Uncertainty. Healthcare. 8. Oktober 2019;7(4):114.

20.	Brown VL, James A, Hunleth J, Bradley CS, Farrar JT, Gupta P, u. a. Believing women: a qualitative exploration of provider disbelief and pain dismissal among women with interstitial cystitis/bladder pain syndrome from the MAPP research network. Int Urogynecology J. Januar 2024;35(1):139–48.

21.	Bull C, Goh JY, Warren N, Kisely S. Experiences of individuals presenting to the emergency department for mental health reasons: A systematic mixed studies review. Aust N Z J Psychiatry. Oktober 2024;58(10):839–56.

22.	Cameron N, Kool M, Estévez-López F, López-Chicheri I, Geenen R. The potential buffering role of self-efficacy and pain acceptance against invalidation in rheumatic diseases. Rheumatol Int. Februar 2018;38(2):283–91.

23.	Carter CR. Gaslighting: ALS, anti‐Blackness, and medicine. Fem Anthropol. November 2022;3(2):235–45.

24.	Chan K, Rubtsova AA, Clark CJ. Exploring diagnosis and treatment of premenstrual dysphoric disorder in the U.S. healthcare system: a qualitative investigation. BMC Womens Health. 17. Mai 2023;23(1):272.

25.	Chilton F, Bradley E, Mitchell T. <i>‘Lost time’</i> . Patients with early inflammatory/rheumatoid arthritis and their experiences of delays in Primary Care. Musculoskeletal Care. Dezember 2021;19(4):495–503.

26.	Christensen V, Parker K, Chan LHK, Saxton L, Cottrell E. ‘Never once was I thinking the c‐word’: Parent perspectives on the facilitators and barriers to getting a childhood cancer diagnosis. J Clin Nurs. Juli 2023;32(13–14):3981–94.

27.	Christoffersen V, Tennfjord MK. Younger Women with Lipedema, Their Experiences with Healthcare Providers, and the Importance of Social Support and Belonging: A Qualitative Study. Int J Environ Res Public Health. 20. Januar 2023;20(3):1925.

28.	Claassen-van Dessel N, R. Velzeboer F, C. Van Der Wouden J, Den Boer C, Dekker J, E. Van Der Horst H. Patients’ Perspectives On Improvement Of Medically Unexplained Physical Symptoms:a Qualitative Analysis. Ment Health Fam Med [Internet]. 30. November 2015 [zitiert 7. November 2025];11(02). Verfügbar unter: http://mhfmjournal.com/open-access/patients-perspectives-on-improvement-of-medically-unexplained-physical-symptoms-a-qualitative-analysis.pdf

29.	Cline RJW, Harper FWK, Penner LA, Peterson AM, Taub JW, Albrecht TL. Parent communication and child pain and distress during painful pediatric cancer treatments. Soc Sci Med. August 2006;63(4):883–98.

30.	Coady A, Godard R, Holtzman S. Understanding the link between pain invalidation and depressive symptoms: The role of shame and social support in people with chronic pain. J Health Psychol. Januar 2024;29(1):52–64.

31.	Corry DAS, Leavey G. Adolescent trust and primary care: Help‐seeking for emotional and psychological difficulties. J Adolesc. Januar 2017;54(1):1–8.

32.	Côté CI. A critical and systematic literature review of epistemic justice applied to healthcare: recommendations for a patient partnership approach. Med Health Care Philos. September 2024;27(3):455–77.

33.	J Davis S. The enormous problem of medical gaslighting. MOJ Addict Med Ther. 7. Oktober 2022;7(1):4–5.

34.	De León-Menjivar C. Understanding the Dialogical Experiences of Puerto Rican Women With Fibromyalgia: An Intersectional Analysis. Hisp Health Care Int. September 2022;20(3):195–201.

35.	Dekker RL, Morton CH, Singleton P, Lyndon A. Women’s Experiences Being Diagnosed With Peripartum Cardiomyopathy: A Qualitative Study. J Midwifery Womens Health. Juli 2016;61(4):467–73.

36.	De Ruddere L, Goubert L, Stevens M, De C. Williams AC, Crombez G. Discounting pain in the absence of medical evidence is explained by negative evaluation of the patient. Pain. Mai 2013;154(5):669–76.

37.	Dieppe P, Goldingay S, Greville-Harris M. The power and value of placebo and nocebo in painful osteoarthritis. Osteoarthritis Cartilage. November 2016;24(11):1850–7.

38.	Durbhakula S, Fortin AH. Turning Down the Flame on Medical Gaslighting. J Gen Intern Med. November 2023;38(15):3426–7.

39.	Eder C, Roomaney R. Transgender and non-binary people’s perception of their healthcare in relation to endometriosis. Int J Transgender Health. Oktober 2024;25(4):911–25.

40.	Edlund SM, Wurm M, Holländare F, Linton SJ, Fruzzetti AE, Tillfors M. Pain patients’ experiences of validation and invalidation from physicians before and after multimodal pain rehabilitation: Associations with pain, negative affectivity, and treatment outcome. Scand J Pain. 1. Oktober 2017;17(1):77–86.

41.	Edlund SM, Söderstrand JC, Åkerman B, Hed E, Larsson M, Tillfors M, u. a. Facing negative emotions: Evaluation of a brief training in validating communication for contact nurses in cancer care. Eur J Oncol Nurs. Oktober 2023;66:102401.

42.	Edmond SN, Keefe FJ. Validating pain communication: current state of the science. Pain. Februar 2015;156(2):215–9.

43.	Ellis K, Munro D, Wood R. Dismissal informs the priorities of endometriosis patients in New Zealand. Front Med. 1. Juni 2023;10:1185769.

44.	Eriksen AA, Fegran L, Fredwall TE, Larsen IB. Patients’ negative experiences with health care settings brought to light by formal complaints: A qualitative metasynthesis. J Clin Nurs. September 2023;32(17–18):5816–35.

45.	Fagen JL, Shelton JA, Luché-Thayer J. Medical Gaslighting and Lyme Disease: The Patient Experience. Healthcare. 29. Dezember 2023;12(1):78.

46.	Ferguson N, Savic M, McCann TV, Emond K, Sandral E, Smith K, u. a. “I was worried if I don’t have a broken leg they might not take it seriously”: Experiences of men accessing ambulance services for mental health and/or alcohol and other drug problems. Health Expect. Juni 2019;22(3):565–74.

47.	Fielding-Singh P, Dmowska A. Obstetric gaslighting and the denial of mothers’ realities. Soc Sci Med. Mai 2022;301:114938.

48.	Frostholm L, Fink P, Oernboel E, Christensen KS, Toft T, Olesen F, u. a. The Uncertain Consultation and Patient Satisfaction: The Impact of Patientsʼ Illness Perceptions and a Randomized Controlled Trial on the Training of Physiciansʼ Communication Skills: Psychosom Med. November 2005;67(6):897–905.

49.	Garcia LM, Jones J, Scandlyn J, Thumm EB, Shabot SC. The meaning of obstetric violence experiences: A qualitative content analysis of the Break the Silence Campaign. Int J Nurs Stud. Dezember 2024;160:104911.

50.	Geenen R. Educating Patients and the Struggle for Validation of Fibromyalgia Syndrome: The Dutch Way. J Musculoskelet Pain. Januar 2009;17(1):80–5.

51.	Gertsman S, Ene IC, Palmert S, Liu A, Makkar M, Shao I, u. a. Clinical empathy as perceived by patients with chronic illness in Canada: a qualitative focus group study. CMAJ Open. September 2023;11(5):E859–68.

52.	Ghavidel-Parsa B, Bidari A, Tohidi S, Shenavar I, Kazemnezhad Leyli E, Hosseini K, u. a. Implication of invalidation concept in fibromyalgia diagnosis. Clin Rheumatol. Juni 2021;40(6):2369–76.

53.	Ghavidel-Parsa B, Bidari A, Rahimi A, Gharibpoor F, Khosousi MJ. No effect of approved fibromyalgia drugs on the social pain (invalidation) contrary to physical pain: an open-label short-term randomized clinical trial. Clin Rheumatol. Januar 2022;41(1):245–54.

54.	Gillie G, Bokek‐Cohen Y. Why are nurses indifferent to the phenomenon of gaslighting of patients in medical systems? Nurs Inq. Oktober 2024;31(4):e12669.

55.	Godman H. Harvard Health. 2024 [zitiert 7. November 2025]. What to do about medical gaslighting. Verfügbar unter: https://www.health.harvard.edu/staying-healthy/what-to-do-about-medical-gaslighting

56.	Goff BA, Mandel L, Muntz HG, Melancon CH. Ovarian carcinoma diagnosis: Results of a national ovarian cancer survey. Cancer. 15. November 2000;89(10):2068–75.

57.	Greville-Harris M, Dieppe P. Bad Is More Powerful than Good: The Nocebo Response in Medical Consultations. Am J Med. Februar 2015;128(2):126–9.

58.	Grundström H, Alehagen S, Kjølhede P, Berterö C. The double‐edged experience of healthcare encounters among women with endometriosis: A qualitative study. J Clin Nurs. Januar 2018;27(1–2):205–11.

59.	Grundström H, Engman L, Rimhagen E, Söderstierna C, Flink I. Experiences of communication in women with endometriosis: perceived validation and invalidation in different contexts, and associations with health-related quality of life. J Psychosom Obstet Gynecol. 31. Dezember 2023;44(1):2264483.

60.	Gunning JN. “But you don’t look sick:” Memorable messages of emerging adulthood autoimmune disease. J Soc Pers Relatsh. Juni 2023;40(6):2008–30.

61.	Haraldsson J, Johnsson L, Tindberg Y, Kristiansson P, Nordgren L. They are my worries, so it’s me the doctor should listen to—adolescent males’ experiences of consultations with general practitioners. BMC Prim Care. 17. Mai 2024;25(1):169.

62.	Hausmann JS, Lomax KG, Shapiro A, Durrant K. The patient journey to diagnosis and treatment of autoinflammatory diseases. Orphanet J Rare Dis. Dezember 2018;13(1):156.

63.	Hearn M, Whorwell PJ, Vasant DH. Stigma and irritable bowel syndrome: a taboo subject? Lancet Gastroenterol Hepatol. Juni 2020;5(6):607–15.

64.	Henry C, Filoche S. Reflections on access to care for heavy menstrual bleeding: Past, present, and in times of the COVID‐19 pandemic. Int J Gynecol Obstet. August 2023;162(S2):23–8.

65.	Herrera A, Leiva L, Espinoza I, Ríos-Erazo M, Shakhtur N, Wurmann P, u. a. Invisible, Uncontrollable, Unpredictable: Illness Experiences in Women with Sjögren Syndrome. J Clin Med. 30. Mai 2024;13(11):3228.

66.	Hildenbrand GM, Perrault EK, Rnoh RH. Patients’ Perceptions of Health Care Providers’ Dismissive Communication. Health Promot Pract. September 2022;23(5):777–84.

67.	Hintz EA. “It’s All in Your Head”: A Meta-Synthesis of Qualitative Research About Disenfranchising Talk Experienced by Female Patients with Chronic Overlapping Pain Conditions. Health Commun. 19. September 2023;38(11):2501–15.

68.	Holopainen R, Lausmaa M, Edlund S, Carstens-Söderstrand J, Karppinen J, O’Sullivan P, u. a. Physiotherapists’ validating and invalidating communication before and after participating in brief cognitive functional therapy training. Test of concept study. Eur J Physiother. 4. März 2023;25(2):73–9.

69.	Houwen J, Lucassen PL, Stappers HW, Assendelft WJ, Van Dulmen S, Hartman TCO. Improving GP communication in consultations on medically unexplained symptoms: a qualitative interview study with patients in primary care. Br J Gen Pract. Oktober 2017;67(663):e716–23.

70.	Houwen J, Lucassen PLBJ, Stappers HW, Assendelft PJJ, Van Dulmen S, Olde Hartman TC. Medically unexplained symptoms: the person, the symptoms and the dialogue. Fam Pract. 24. Januar 2017;cmw132.

71.	Hutchens J, Frawley J, Sullivan EA. Is self-advocacy universally achievable for patients? The experiences of Australian women with cardiac disease in pregnancy and postpartum. Int J Qual Stud Health Well-Being. 31. Dezember 2023;18(1):2182953.

72.	Jannesari A. BIPOC Identity, Delayed Diagnosis, Pain, and Medical Gaslighting as Predictors of Post-traumatic Stress Symptomatology in Adults With Endometriosis [Internet] [Psy.D.]. ProQuest Dissertations and Theses. [United States -- California]: Alliant International University; 2024 [zitiert 7. November 2025]. Verfügbar unter: https://www.proquest.com/docview/3094651637/abstract/D3569AC7A2B34F3EPQ/1

73.	Järemo P, Arman M, Gerdle B, Gottberg K. Facing Invalidation: A Further Challenge when Living with Chronic Widespread Pain. J Rehabil Med. 22. April 2022;54:jrm00280.

74.	Jedick R. Human Factors in Medicine: Medical Gaslighting or a Broken Health Care System? Emerg Med News. Oktober 2022;44(10):23–23.

75.	Katz SJ. Gender Disparities in the Receipt of Home Care for Elderly People With Disability in the United States. JAMA. 20. Dezember 2000;284(23):3022.

76.	Kauppi W, Axelsson C, Herlitz J, Jiménez‐Herrera M, Palmér L. Lived experiences of being cared for by ambulance clinicians when experiencing breathlessness—A phenomenological study. Scand J Caring Sci. März 2023;37(1):207–15.

77.	Kendrick EA, Beesley D. Perceived stress, illness invalidation, and symptom severity in myalgic encephalomyelitis/chronic fatigue syndrome. Fatigue Biomed Health Behav. 1. Oktober 2016;4(4):217–25.

78.	Khan K, Tariq NUS, Majeed S. Psychological Impact of Medical Gaslighting on Women: A Systematic Review. J Prof Appl Psychol. 21. März 2024;5(1):110–25.

79.	Kieren MQ, Kelly MM, Garcia MA, Chen T, Ngo T, Baird J, u. a. Parent Experiences with the Process of Sharing Inpatient Safety Concerns for Children with Medical Complexity: A Qualitative Analysis. Acad Pediatr. November 2023;23(8):1535–41.

80.	King SR, Allan M, Lindsey L. “I found hundreds of other people…but I still wasn’t believed” – An exploratory study on lived experiences of antipsychotic withdrawal. Psychosis. 2. Januar 2024;16(1):15–27.

81.	Kool MB, Van Middendorp H, Lumley MA, Schenk Y, Jacobs JWG, Bijlsma JWJ, u. a. Lack of understanding in fibromyalgia and rheumatoid arthritis: the Illness Invalidation Inventory (3*I). Ann Rheum Dis. November 2010;69(11):1990–5.

82.	Stone L. Making sense of medically unexplained symptoms in general practice: a grounded theory study. Ment Health Fam Med. Juni 2013;10(2):101–11.

83.	Kool MB, Geenen R. Loneliness in Patients with Rheumatic Diseases: The Significance of Invalidation and Lack of Social Support. J Psychol. Januar 2012;146(1–2):229–41.

84.	Kool MB, Middendorp HV, Bijlsma JWJ, Geenen R. Patient and spouse appraisals of health status in rheumatoid arthritis and ﬁbromyalgia: discrepancies and associations with invalidation. 2011;

85.	Kool MB, Van De Schoot R, López-Chicheri García I, Mewes R, Da Silva JAP, Vangronsveld K, u. a. Measurement invariance of the Illness Invalidation Inventory (3*I) across language, rheumatic disease and gender. Ann Rheum Dis. März 2014;73(3):551–6.

86.	Laestadius LI, Guidry JPD, Wahl MM, Perrin PB, Carlyle KE, Dong X, u. a. “The dream is that there’s one place you go”: a qualitative study of women’s experiences seeking care from Long COVID clinics in the USA. BMC Med. 13. Juni 2024;22(1):243.

87.	Larrea S, Assis MP, Mendoza CO. “Hospitals have some procedures that seem dehumanising to me”: Experiences of abortion-related obstetric violence in Brazil, Chile and Ecuador. Agenda. 3. Juli 2021;35(3):54–68.

88.	Lesnewich LM, Hyde JK, McFarlin ML, Bolton RE, Bayley PJ, Chandler HK, u. a. ‘She thought the same way I that I thought:’ a qualitative study of patient-provider concordance among Gulf War Veterans with Gulf War Illness. Psychol Health. 3. April 2025;40(4):616–34.

89.	Lian OS, Robson C. “It´s incredible how much I´ve had to fight.” Negotiating medical uncertainty in clinical encounters. Int J Qual Stud Health Well-Being. Januar 2017;12(1):1392219.

90.	Liggins J, Hatcher S. Stigma toward the mentally ill in the general hospital: a qualitative study. Gen Hosp Psychiatry. September 2005;27(5):359–64.

91.	Lightbourne A, Foley S, Dempsey M, Cronin M. Living With Endometriosis: A Reflexive Thematic Analysis Examining Women’s Experiences With the Irish Healthcare Services. Qual Health Res. März 2024;34(4):311–22.

92.	Linton SJ, Flink IK, Nilsson E, Edlund S. Can training in empathetic validation improve medical students’ communication with patients suffering pain? A test of concept. PAIN Rep. Mai 2017;2(3):e600.

93.	Lobo CP, Pfalzgraf AR, Giannetti V, Kanyongo G. Impact of Invalidation and Trust in Physicians on Health Outcomes in Fibromyalgia Patients. Prim Care Companion CNS Disord [Internet]. 9. Oktober 2014 [zitiert 6. November 2025]; Verfügbar unter: https://www.psychiatrist.com/pcc/impact-invalidation-trust-physicians-health-outcomes

94.	López-Aybar L, Gonzales L, Kanani A. Prosumers’ experiences of stigma dimensions within the clinical psychology field. Psychol Serv. Mai 2024;21(2):369–78.

95.	Lund T, Neville A, Jordan A, Carter B, Sumpton J, Noel M. Narrative Accounts of Youth and Their Mothers With Chronic Headache: Application of a Socio-narratology Framework to Pain Narratives. Clin J Pain. Oktober 2024;40(10):588–600.

96.	Mathur VA, Payano Sosa JS, Keaser ML, Meiller TF, Seminowicz DA. The social context of burning mouth syndrome: an exploratory pilot study of stigma, discrimination, and pain. Pain Med. 2. November 2023;24(11):1213–8.

97.	Molin B, Zwedberg S, Berger AK, Sand A, Georgsson S. “The ignored pain” - experiences of encounters with healthcare from the perspective of women with pain persisting after childbirth - a qualitative study. Sex Reprod Healthc. März 2024;39:100929.

98.	Mullard J, Mir G, Herbert C, Evans S, LOCOMOTION Consortium. ‘You’re just a Guinea pig’: Exploring the barriers and impacts of living with long COVID‐19: A view from the undiagnosed. Sociol Health Illn. November 2024;46(8):1602–25.

99.	Munro M, Cook AM, Bogart KR. An inductive qualitative content analysis of stigma experienced by people with rare diseases. Psychol Health. 3. August 2022;37(8):948–63.

100.	Newsom KD, Riddle MJ, Carter GA, Hille JJ. They “Don’t Know How to Deal with People Like Me”: Assessing Health Care Experiences of Gender Minorities in Indiana. Transgender Health. 1. Oktober 2022;7(5):453–60.

101.	Ng IK, Tham SZ, Singh GD, Thong C, Teo DB. Medical Gaslighting: A New Colloquialism. Am J Med. Oktober 2024;137(10):920–2.

102.	Nicola M, Correia H, Ditchburn G, Drummond PD. Defining pain-validation: The importance of validation in reducing the stresses of chronic pain. Front Pain Res. 14. Oktober 2022;3:884335.

103.	Nicola M, Correia H, Ditchburn G, Drummond PD. The Pain-Invalidation Scale: Measuring Patient Perceptions of Invalidation Toward Chronic Pain. J Pain. November 2022;23(11):1912–22.

104.	Nicola M, Correia H, Ditchburn G, Drummond P. Invalidation of chronic pain: a thematic analysis of pain narratives. Disabil Rehabil. 13. März 2021;43(6):861–9.

105.	Niedenfuehr J, Edwards M, King LM. A scoping review: the psychosocial barriers that exist for people with vulvodynia. J Sex Med. 26. Mai 2023;20(6):833–58.

106.	Nishikawara RK, Schultz IZ, Butterfield LD, Murray JW. “You have to believe the patient”: What do people with fibromyalgia find helpful (and hindering) when accessing health care? Can J Pain. 30. Juli 2023;7(2):2176745.

107.	O’Malley G, Irwin L, Guerin S. Supporting People with Intellectual Disability Who Have Experienced Abuse: Clinical Psychologists’ Perspectives. J Policy Pract Intellect Disabil. März 2020;17(1):59–69.

108.	Oosterhof B, Dekker JHM, Sloots M, Bartels EAC, Dekker J. Success or failure of chronic pain rehabilitation: the importance of good interaction – a qualitative study under patients and professionals. Disabil Rehabil. Oktober 2014;36(22):1903–10.

109.	Owen R, Ashton RE, Skipper L, Phillips BE, Yates J, Thomas C, u. a. Long COVID quality of life and healthcare experiences in the UK: a mixed method online survey. Qual Life Res. Januar 2024;33(1):133–43.

110.	Patel H, Schaufelberger M, Begley C, Berg M. Experiences of health care in women with Peripartum Cardiomyopathy in Sweden: a qualitative interview study. BMC Pregnancy Childbirth. Dezember 2016;16(1):386.

111.	Plackowski EF, Bogart KR. “If not me, who?”: Awareness- and Self-Advocacy-Related Experiences of Adults With Diverse Rare Disorders. Qual Health Res. Januar 2023;33(1–2):63–80.

112.	Price J, Farmer G, Harris J, Hope T, Kennedy S, Mayou R. Attitudes of women with chronic pelvic pain to the gynaecological consultation: a qualitative study. BJOG Int J Obstet Gynaecol. April 2006;113(4):446–52.

113.	Qureshi AG, Jha SK, Iskander J, Avanthika C, Jhaveri S, Patel VH, u. a. Diagnostic Challenges and Management of Fibromyalgia. Cureus [Internet]. 11. Oktober 2021 [zitiert 6. November 2025]; Verfügbar unter: https://www.cureus.com/articles/73825-diagnostic-challenges-and-management-of-fibromyalgia

114.	Rebman AW, Yang T, Aucott JN. Invalidation by medical professionals in post-treatment Lyme disease. Sci Rep. 21. August 2024;14(1):19406.

115.	Renault NK, Howell RE, Robinson KS, Greer WL. Qualitative assessment of the emotional and behavioural responses of haemophilia A carriers to negative experiences in their medical care: HA CARRIER RESPONSES TO THEIR MEDICAL CARE. Haemophilia. März 2011;17(2):237–45.

116.	Riggs DW, Bartholomaeus C. Gaslighting in the context of clinical interactions with parents of transgender children. Sex Relatsh Ther. 2. Oktober 2018;33(4):382–94.

117.	Russell D, Spence NJ, Chase JAD, Schwartz T, Tumminello CM, Bouldin E. Support amid uncertainty: Long COVID illness experiences and the role of online communities. SSM - Qual Res Health. Dezember 2022;2:100177.

118.	Sakalys JA. Illness behavior in rheumatoid arthritis. Arthritis Rheum. August 1997;10(4):229–37.

119.	Salmon P. Conflict, collusion or collaboration in consultations about medically unexplained symptoms: The need for a curriculum of medical explanation. Patient Educ Couns. August 2007;67(3):246–54.

120.	Samsson KS, Bernhardsson S, Larsson ME. “Take me seriously and do something!” - a qualitative study exploring patients’ perceptions and expectations of an upcoming orthopaedic consultation. BMC Musculoskelet Disord. Dezember 2017;18(1):367.

121.	Samulowitz A, Gremyr I, Eriksson E, Hensing G. “Brave Men” and “Emotional Women”: A Theory-Guided Literature Review on Gender Bias in Health Care and Gendered Norms towards Patients with Chronic Pain. Pain Res Manag. 2018;2018:1–14.

122.	Santiago MG, Marques A, Kool M, Geenen R, Da Silva JAP. Invalidation in Patients with Rheumatic Diseases: Clinical and Psychological Framework. J Rheumatol. April 2017;44(4):512–8.

123.	Schäfer G, Prkachin KM, Kaseweter KA, Williams ACDC. Health care providers’ judgments in chronic pain: the influence of gender and trustworthiness. Pain. August 2016;157(8):1618–25.

124.	Sebring JCH. Towards a sociological understanding of medical gaslighting in western health care. Sociol Health Illn. November 2021;43(9):1951–64.

125.	Sebring JCH, Kelly C, McPhail D, Woodgate RL. Medical invalidation in the clinical encounter: a qualitative study of the health care experiences of young women and nonbinary people living with chronic illnesses. CMAJ Open. September 2023;11(5):E915–21.

126.	Sells D, Black R, Davidson L, Rowe M. Beyond Generic Support: Incidence and Impact of Invalidation in Peer Services for Clients With Severe Mental Illness. Psychiatr Serv. November 2008;59(11):1322–7.

127.	Sharpe M. Medical gaslighting: to say that invoking psychological symptoms is equivalent to dismissal is reductionist. BMJ. 15. September 2022;o2236.

128.	Sheikhan NY, Henderson JL, Halsall T, Daley M, Brownell S, Shah J, u. a. Stigma as a barrier to early intervention among youth seeking mental health services in Ontario, Canada: a qualitative study. BMC Health Serv Res. 26. Januar 2023;23(1):86.

129.	Singh G, Hamid A. Invalidation in fibromyalgia and rheumatoid arthritis and its effect on quality of life in Indian patients. Int J Rheum Dis. August 2021;24(8):1047–52.

130.	Sloan M, Bosley M, Blane M, Holloway L, Barrere C, D’Cruz D, u. a. ‘But you don’t look sick’: a qualitative analysis of the LUPUS UK online forum. Rheumatol Int. April 2021;41(4):721–32.

131.	Sloan M, Harwood R, Sutton S, D’Cruz D, Howard P, Wincup C, u. a. Medically explained symptoms: a mixed methods study of diagnostic, symptom and support experiences of patients with lupus and related systemic autoimmune diseases. Rheumatol Adv Pract. 1. Januar 2020;4(1):rkaa006.

132.	Soucie K, Tapp K, Kobrosli J, Rakus M, Katzman R, Schramer K, u. a. “It Wasn’t Until I Took the Reins and Said….” Power and Advocacy in Canadian Women’s Narratives of Polycystic Ovary Syndrome Diagnosis and Treatment*. Womens Reprod Health. 2. Januar 2023;10(1):88–109.

133.	Staal AHJ, Van Der Zanden M, Nap AW. Diagnostic Delay of Endometriosis in the Netherlands. Gynecol Obstet Invest. 2016;81(4):321–4.

134.	Sword W, Busser D, Ganann R, McMillan T, Swinton M. Women’s Care-Seeking Experiences After Referral for Postpartum Depression. Qual Health Res. September 2008;18(9):1161–73.

135.	Tang S, Anderson NE, Faasse K, Adams WP, Newby JM. A Qualitative Study on the Experiences of Women With Breast Implant Illness. Aesthet Surg J. 15. März 2022;42(4):381–93.

136.	Nogrady B. How pain is misunderstood and ignored in women. Nature. 26. September 2024;633(8031):S31–3.

137.	Thompson CM, Babu S, Makos S. Women’s Experiences of Health-Related Communicative Disenfranchisement. Health Commun. 6. Dezember 2023;38(14):3135–46.

138.	Thompson CM, Babu S, Makos S. A Grounded Theory of Credibility Work and Illness: Explication and Application to the Case of Women on Trial in Health Care. Commun Res. Dezember 2025;52(8):1059–85.

139.	Tjernström K, Lindberg I, Wiklund M, Persson M. Overlooked by the obstetric gaze – how women with persistent health problems due to severe perineal trauma experience encounters with healthcare services: a qualitative study. BMC Health Serv Res. 9. Mai 2024;24(1):610.

140.	Treufeldt H, Burton C. Stigmatisation in medical encounters for persistent physical symptoms/functional disorders: Scoping review and thematic synthesis. Patient Educ Couns. Juni 2024;123:108198.

141.	Kool MB, Van Middendorp H, Boeije HR, Geenen R. Understanding the lack of understanding: Invalidation from the perspective of the patient with fibromyalgia. Arthritis Care Res. 15. Dezember 2009;61(12):1650–6.

142.	Vangronsveld KL, Linton SJ. The effect of validating and invalidating communication on satisfaction, pain and affect in nurses suffering from low back pain during a semi‐structured interview. Eur J Pain. Februar 2012;16(2):239–46.

143.	Verbeek JH, De Boer AG, Van Der Weide WE, Piirainen H, Anema JR, Van Amstel RJ, u. a. Patient satisfaction with occupational health physicians, development of a questionnaire. Occup Environ Med. Februar 2005;62(2):119–23.

144.	Vriezekolk JE, Peters AJF, Van Den Ende CHM, Geenen R. Solicitous and invalidating responses are associated with health-care visits in fibromyalgia. Rheumatol Adv Pract. 1. Januar 2019;3(1):rkz008.

145.	Walkden SM. “So It’s Like a Painful Period?” Living with Endometriosis: My Journey. Health Commun. 14. Oktober 2024;39(12):2950–4.

146.	Wasson Simpson KS, Gallagher A, Ronis ST, Miller DAA, Tilleczek KC. Youths’ Perceived Impact of Invalidation and Validation on Their Mental Health Treatment Journeys. Adm Policy Ment Health Ment Health Serv Res. Mai 2022;49(3):476–89.

147.	Werner A, Malterud K. It is hard work behaving as a credible patient: encounters between women with chronic pain and their doctors. Soc Sci Med. Oktober 2003;57(8):1409–19.

148.	Wise J. Sixty seconds on . . . medical gaslighting. BMJ. 9. August 2022;o1974.

149.	Yadava OP. ‘Medical gaslighting’—an eye-opener. Indian J Thorac Cardiovasc Surg. September 2023;39(5):441–2.

150.	Zugai JS, Gill K, Molloy L, Raeburn T, Ramjan L. The focus on weight in the inpatient care of anorexia nervosa: A qualitative investigation of consumer perspectives. Int J Ment Health Nurs. April 2024;33(2):388–96.
