# Supplementary material for: What do we know about medical invalidation and related concepts? – A scoping review and thematic analysis about the definitions, measurements, causes, consequences and potential solutions for medical invalidation
Source: BMC Health Serv Res. 2026 Sep 8;26:1252. doi: 10.1186/s12913-026-14736-3 (PMC13560384; doi:10.1186/s12913-026-14736-3)
Supplement: Supplementary file 6 [file 12913_2026_14736_MOESM6_ESM.docx]

| **Main themes** | **Subthemes** | **Subsubthemes** | **Subsubsubthemes** | **Description** |
| --- | --- | --- | --- | --- |
| **Diagnostic**  **process** | Long diagnostic processes |  |  | This theme describes that long diagnostic processes (either necessary due to characteristics of the disease or characteristics of the health care system) contributes to the experience of invalidation. |
|  | Diagnostic criteria | Missing of subjective and difficult to quantify symptoms within diagnostic criteria |  | Some of acknowledged diagnostic criteria might me to vague for the implementation into praxis and therefore not useable, contributing to invalidation. |
|  |  | Inconsistent diagnostic criteria |  | Some of acknowledged diagnostic criteria might be inconsistent and therefore contributing to invalidation. |
|  |  | Lack of a formal diagnostic code |  | For some symptom complexes, diseases, or similar, a formal diagnostic code is still lacking. |
|  | Lack of objective diagnostic tests |  |  | If diagnostic tests are not invented it or not available due to different reasons. |
|  | | | | |
| **Societal discourses / cultural norms** | Cultural expectations |  |  | Cultural expectations for example include that every illness, symptoms or similar should have a label and treatment or is explainable by the biomedical model. |
|  | Cultural injustice |  |  | Health disparities between different cultural background might foster invalidation. |
|  | Societal pressures of idealism |  |  | The cultural norm that doctors are placed on a godly pedestal and are hard to question contributes to invalidation. |
|  | Partriarchal influence and Policies |  |  | Invalidation, especially of women, can be explained through patriarchal influences and policies. |
|  | People with intellectual disability being treated as inferior |  |  | The credibility of a disabled atient can be questioned if the cultural norm sees people with disability as inferior. |
|  | Societal beliefs about severity threshold |  |  | If there are societal beliefs about a severity threshold in a disease or symptom, this can lead to both, invalidation of HCP or self-invalidation. |
|  | Expectations of women |  |  | Because medicine has historically treated women’s bodies and symptoms as less credible, women are often expected to be uncertain or passive about their own health. These gendered expectations make it easier for healthcare professionals to dismiss women’s reports, reinforcing a pattern in which women’s concerns are questioned or minimized far more often than men’s |
|  | | | | |
| **Structual / Systemic issues** | Economic burden | Increasingly unhealthy patient population |  | Systemic contributors included economic burdens such as restraints by insurances or more administrative work leading to fewer time for patients. An increasingly unhealthy patient-population also contributes to increased need of resources and an economic burden. |
|  |  | Administration |  |  |
|  |  | Restraints by insurance |  |  |
|  | Systemic reductionism in Western medicine/ Biomedical disease model |  |  | This refers to the tendency of biomedical systems to reduce complex, multidimensional human experiences — such as pain, fatigue, or psychological suffering — to narrowly defined physiological parameters. This reductionist framework often prioritizes measurable, objective indicators over patients’ subjective accounts, thereby invalidating symptoms or experiences that cannot be easily quantified or medically explained. |
|  | Epistemic injustice |  |  | Epistemic injustice occurs when a person’s knowledge or account of their own experience is undervalued, leading healthcare professionals to overlook or dismiss their concerns. |
|  | Pressure on staff… | High workload |  | High workload within the system might but strain and pressure on staff, make it more likely for invalidation to occur. |
|  |  | High demand on communication skills |  | Complex and complicated situations (e.g. medical uncertainty) might put a high demand on communication skills on staff, that not all achieve. |
|  | Healthcare access barriers |  |  | Gatekeepers within health services (e.g. primary care doctors, insurance applications or payment systems) should be considered as contributors to invalidation. |
|  | Lack of… | Ressources |  | A general lack of resources within the health care system contributes to the experience of invalidation. |
|  |  | Time |  | A general lack of time within the health care system contributes to the experience of invalidation. |
|  |  | Training | General training (competence) | A general lack of training to obtain certain competencies contributes to the experience of invalidation. |
|  |  |  | Medicine is not caring about intuition and empathy | A general lack of training for interpersonal and communication skills contributes to the experience of invalidation. |
|  |  | Guidelines |  | A lack of specific guidelines for diseases or processes within health care contributes to the experience of invalidation. |
|  | | | | |
| **Misattribution of symptoms** | Misdiagnosis… | to psychological / psychiatric causes / diagnostic overshadowing |  | There is a risk of prematurely concluding that a mental illness is present when symptoms overlap, especially when personal characteristics are present or disease-specific characteristics are present. This includes to attribute symptoms to an already known psychological disease in the patient instead of acknowledging there could be a newly emerged (somatic) problem (diagnostic overshadowing). |
|  | | | | |
| **Interaction between patient and medical personal** | Lack of conversations |  |  | A lack of conversation despite the necessity between patient-provider is a reason for the experience of invalidation. |
|  | Insufficient treatment offer |  |  | The patient receiving an insufficient treatment offer due to several reasons might lead to the feeling of invalidation. |
|  | Frequent visits to a doctor |  |  | Frequent visits to a doctor despite the reasons (e.g. already having many chronic conditions) contributes to invalidation. |
|  | Power imbalance |  |  | Power imbalances between patient-provider contributes to the experience of invalidation. |
|  | Limited understanding due to mismatch of perspective |  |  | Limited understanding for each other (e.g. perspective taking of life situation or lack of knowledge of the disease) contributes to invalidation. |
|  | Disparate health beliefs leading to not accepting the physician´s explanation |  |  | If patients do not accept the physicians explanation due to the lack of consideration of the patients subjective illness behaviour, invalidation is risked. |
|  | Communication problems |  |  | Communication problems between patient-and provider contribute to invalidation. |
|  | | | | |
| **Academic research** | Underresearched medical topics leading to medical uncertainty |  |  | Lack of knowledge often goes hand in hand with medical uncertainty, which in itself is a factor contributing to invalidation. |
|  | Lack of sex-specific research |  |  | Due to a lack of sex-specific research, there is limited knowledge of sex specific symptoms, a “gender data gap”, which contributes to invalidation. |
|  | Lack of research to transgender health |  |  | Due to a lack of research in the field of transgender health, little knowledge is gathered in this area, which in turn increases likelihood for invalidation for transgender people. |
|  | Bias in research |  |  | Some research is biased, and if this is the case, it affects patient care and contributes to invalidation due to a lack of knowledge. |
|  | | | | |
| **Stigma** | Conscious or unconscious bias |  |  | Deliberate (conscious) or automatic (unconscious) prejudicial attitudes or assumptions that influence judgment. Such biases can lead clinicians to dismiss or minimize patients’ symptoms, contributing directly to medical invalidation. |
|  | Menstrual stigma |  |  | Negative cultural beliefs, taboos, and stereotypes surrounding menstruation that frame it as abnormal, exaggerated, or pathological. This stigma often results in healthcare providers trivializing menstrual pain or symptoms, thereby reinforcing medical invalidation of menstruators. |
|  | Interpersonally enacted stigmas |  |  | Stigmatizing behaviors expressed directly between individuals, such as dismissive comments or differential treatment. In clinical encounters, these behaviors can cause patients to feel disbelieved or delegitimized, fostering medical invalidation. |
|  | Awareness bias |  |  | A cognitive bias in which individuals underestimate or overlook conditions or experiences outside their own awareness or expertise. In healthcare, this can lead practitioners to dismiss patient reports that do not align with their expectations, contributing to invalidation. |
|  | Implicit bias |  |  | Automatic, unconscious associations or stereotypes that influence perception and behavior without deliberate intent. Implicit biases can cause clinicians to discount patient reports—especially from marginalized groups—thereby perpetuating medical invalidation. |
|  | Facial-based trustworthiness heuristics |  |  | Rapid, automatic judgments of trustworthiness based on facial features, often unrelated to actual credibility. Healthcare providers may unconsciously deem certain patients less trustworthy, increasing the likelihood of dismissing their symptoms and producing medical invalidation. |
|  | Internalized stigma |  |  | The process by which individuals absorb and accept negative societal stereotypes about their own condition or identity. Patients experiencing internalized stigma may minimize or doubt their own symptoms, making them more vulnerable to medical invalidation. |
|  | Social stigma |  |  | Broad societal devaluation of certain identities, conditions, or experiences that shapes norms and institutional practices. Social stigma can influence clinical decision-making, leading to systemic patterns of dismissing or discrediting patients’ health concerns. |
|  | | | | |
| **Characteristics of the disease** | Psychological comorbidities or symptoms within a somatic disease |  |  | If there are psychological comorbidities or a somatic disorder presents with psychological symptoms, psychological aspects are often taken as an explainable reason for the symptoms instead of thoroughly investigating the symptoms. |
|  |  | Complex symptoms/more reported symptoms |  | Very complex symptoms make it harder to diagnose the patient, which in turn contributes to invalidation. |
|  |  | Non-specific / ambigious symptoms |  | Many diseases have non-specific symptoms (e.g. tiredness), which can contribute to invalidation in various ways. |
|  |  | Attypical symtpoms |  | If patients present with atypical symptoms of a disease, this can make it harder to diagnose, therefore also contributing to invalidation. |
|  |  | Symptoms not viewed holistically |  | If symptoms are not viewed holistically, e.g. one doctor cares for type A of symptoms and another for type B of symptoms, this can lead to invalidation in various ways. |
|  | The disease itself | LUPUS |  | Patients with some specific diseases are more likely to be invalidated. So far this association has been found for Lupus. |
|  |  | Medically unexplained symptoms (MUS) |  | Medically unexplained symptoms are defined as subjective patient symptoms for which the treating physician, other healthcare providers, and research scientists have found no medical cause. If there is no explainable cause, this can give patients the sense of invalidation. The “unexpalinability” may come from different reason, e.g. wrong diagnostic test, lack of research of the disease or others. |
|  |  | Chronic pelvic pain |  | Patients with some specific diseases are more likely to be invalidated. So far this association has been found for chronic pelvic pain. |
|  |  | Lyme Disease |  | Patients with some specific diseases are more likely to be invalidated. So far this association has been found for Lyme Disease. |
|  |  | Rheumatic Diseases |  | Patients with some specific diseases are more likely to be invalidated. So far this association has been found for rhematic diseases. |
|  |  | Pramenstrual disphoric disorder PMDD) |  | Patients with some specific diseases are more likely to be invalidated. So far this association has been found for PMDD. |
|  |  | Inherited bleeding disorders |  | Patients with some specific diseases are more likely to be invalidated. So far this association has been found for inherited bleeding disorders. |
|  |  | Pain patients |  | Patients with some specific diseases are more likely to be invalidated. So far this association has been found for pain patients. |
|  |  | Long Covid |  | Patients with some specific diseases are more likely to be invalidated. So far this association has been found for Long Covid. |
|  |  | Policystic ovarian syndrome (PCOS) |  | Patients with some specific diseases are more likely to be invalidated. So far this association has been found for PCOS |
|  |  | Pregnancy | Postpartal Cardiomiopathy (PPCM) | Pregnant women with associated conditions are more likely to be invalidated. So far this association has been found for PPCM. |
|  |  |  | Postpartum Depression | Pregnant women with associated conditions are more likely to be invalidated. So far this association has been found for postpartum depression. |
|  |  |  | Abortion-related symptoms | Pregnant women with associated conditions are more likely to be invalidated. So far this association has been found for abortion-related symptoms. |
|  |  |  | Postpartum Pain | Pregnant women with associated conditions are more likely to be invalidated. So far this association has been found for postpartum pain. |
|  |  |  | Postpartum women´s physical decreased well-being. | Pregnant women with associated conditions are more likely to be invalidated. So far this association has been found for decreased well-being in postpartum women. |
|  |  | Functional Disorders |  | Patients with some specific diseases are more likely to be invalidated. So far this association has been found for functional disorders. |
|  |  | Fibromyalgia |  | Patients with some specific diseases are more likely to be invalidated. So far this association has been found for fibromyalgia. |
|  |  | Endometriosis |  | Patients with some specific diseases are more likely to be invalidated. So far this association has been found for endometriosis. |
|  | Lack of education/knowledge  and understanding of the disease |  |  | In general, if there is little knowledge or understanding of the disease, this is a risk factor for invalidation to occur. |
|  | | | | |
| **Characteristics of the patients** | Substance abuse |  |  | Patients who report substance abuse are at higher risk of invalidation. |
|  | Prioritizing others over themselfes |  |  | Patients that prioritize others over themselves are more likely to be invalidated. |
|  | Social environment | Support and guidance from others |  | Patients with support and guidance from others, e.g. family members or support groups are more protected from experiencing invalidation. |
|  | Appearance / weight |  |  | Appearance or weight of patients can contribute to invalidation, e.g. throught (weight bias) or misattribution of symptoms. |
|  | Socioeconomic status | No job |  | Lower socioecomic status, e.g. no job, lower education and insurance status might increase the likelihood of invalidation. |
|  |  | Lower education |  |  |
|  |  | Insurance status |  |  |
|  | Intersectionality |  |  | Intersectionality in the experience of invalidation refers to how overlapping social identities—such as gender, race, class, or disability—interact to shape individuals’ likelihood of being dismissed or disbelieved in clinical settings. These intersecting identities compound vulnerability, leading some groups to experience invalidation more frequently or more intensely than others. |
|  | Demographic Factors | Age |  | Some demographic characteristics might contribute to the occurrence of invalidation, e.g. age, race, as well as sex and gender. |
|  |  | Race |  |  |
|  |  | Gender |  |  |
|  |  | Sex |  |  |
|  | Not taking the own symptoms seriously |  |  | Patients who do not take their own symptoms seriously, are more likely to experience invalidation. |
|  | | | | |
| **Characteristics of the people not taking seriously** | Attitude of the HCP |  |  | A healthcare professional’s general disposition—such as being dismissive, skeptical, or patronizing—can directly influence whether patients’ symptoms are taken seriously, contributing to invalidation. |
|  | Limited time |  |  | Time pressure in clinical encounters may lead HCPs to shortcut assessments or overlook patient narratives, increasing the likelihood of prematurely dismissing symptoms. |
|  | HCP´s personal moral stance interferes with quality of healthcare |  |  | When personal beliefs or moral judgments shape clinical decision-making, HCPs may consciously or unconsciously devalue certain patients’ experiences, resulting in invalidation. |
|  | Lack of preparedness |  |  | Insufficient training, knowledge, or diagnostic confidence can cause HCPs to misinterpret or minimize symptoms they do not fully understand. |
|  | Uncomfortable feeling during conversation |  |  | Discomfort with sensitive topics or emotionally charged discussions may lead HCPs to deflect, downplay, or avoid patient concerns, thereby fostering invalidation. |
|  | | | | |
| **Theoretical explanation models** | Focauldian bipower |  |  | Foucauldian biopower helps explain medical invalidation by showing how healthcare systems maintain control through norms about how bodies should behave and how patients should act. When someone’s experience doesn’t fit these medical norms—or when they’re seen as ‘non-compliant’—their reports are more likely to be doubted or dismissed. Invalidation becomes a way for the system to reassert authority and restore compliance. |
|  | Medical habitus and doxa |  |  | Medical habitus and doxa explain invalidation by highlighting how healthcare professionals internalize shared assumptions about what ‘typical’ symptoms, patients, and behaviors look like. When a patient’s account falls outside these unquestioned norms, it can be perceived as less credible. As a result, clinicians may dismiss or downplay experiences that don’t align with their ingrained expectations. |
|  | Dualistic medical model |  |  | The dualistic medical model, which separates mind and body, can lead to invalidation when a patient’s symptoms don’t show clear physical markers. Experiences that can’t be easily located in the body are often dismissed as psychological or ‘not real.’ This split encourages clinicians to minimize conditions that don’t fit a strictly biological framework. |
